# Identification of miRNA-mediated drought responsive multi-tiered regulatory network in drought tolerant rice, Nagina 22

**DOI:** 10.1038/s41598-017-15450-1

**Published:** 2017-11-13

**Authors:** Sonia Balyan, Mukesh Kumar, Roseeta Devi Mutum, Utkarsh Raghuvanshi, Priyanka Agarwal, Saloni Mathur, Saurabh Raghuvanshi

**Affiliations:** 1 Department of Plant Molecular Biology, University of Delhi South Campus, Benito Juarez Road, New Delhi, 110021 India; 2 National Institute of Plant Genome Research, Aruna Asaf Ali Road, New Delhi, 110067 India

## Abstract

Comparative characterization of microRNA-mediated stress regulatory networks in contrasting rice cultivars is critical to decipher plant stress response. Consequently, a multi-level comparative analysis, using sRNA sequencing, degradome analysis, enzymatic and metabolite assays and metal ion analysis, in drought tolerant and sensitive rice cultivars was conducted. The study identified a group of miRNAs “Cultivar-specific drought responsive” (CSDR)-miRNAs (osa-miR159f, osa-miR1871, osa-miR398b, osa-miR408-3p, osa-miR2878-5p, osa-miR528-5p and osa-miR397a) that were up-regulated in the flag-leaves of tolerant cultivar, Nagina 22 (N22) and Vandana, but down-regulated in the sensitive cultivar, Pusa Basmati 1 (PB1) and IR64, during drought. Interestingly, CSDR-miRNAs target several copper-protein coding transcripts like plantacyanins, laccases and Copper/Zinc superoxide dismutases (Cu/Zn SODs) and are themselves found to be similarly induced under simulated copper-starvation in both N22 and PB1. Transcription factor OsSPL9, implicated in Cu-homeostasis also interacted with osa-miR408-3p and osa-miR528-5p promoters. Further, N22 flag leaves showed lower SOD activity, accumulated ROS and had a higher stomata closure. Interestingly, compared to PB1, internal Cu levels significantly decreased in the N22 flag-leaves, during drought. Thus, the study identifies the unique drought mediated dynamism and interplay of Cu and ROS homeostasis, in the flag leaves of drought tolerant rice, wherein CSDR-miRNAs play a pivotal role.

## Introduction

Rice production is severely constrained by drought, especially if encountered during panicle initiation and flowering. Several traditional rice cultivars have inherent tolerance mechanisms functioning in diverse environmental adversities^1,2^. The genetic and molecular characterization of these traditional tolerant germplasm at the level of genome, metabolome, transcriptome, proteome and miRNome is critical to understand the schemas and molecular networks regulating stress tolerance and to identify candidate genes involved in stress tolerance. Drought is a complicated phenomenon involving multiple components wherein a subtle fine-tuning of component(s) can set a chain of events that may offer advantage to the plant during stress. Thus, detailed studies that can identify and integrate information^3^ about such multi-dimensional networks wherein regulatory and metabolic components are critical for understanding complex traits. Subtle variations in the activity of any of the components of the network would have a cascading affect on the entire network. Plants have evolved several different strategies to counter stress conditions including root exploration, water conservation, osmotic adjustment, root-soil isolation, leaf orientation, root to shoot ratio, stress recovery^4^. MiRNAs define one such dimension since they have been implicated in regulation of diverse biological processes such as response to abiotic^5–9^ and biotic^10^ stress response, development^11–13^, hormone signaling^14,15^ and nutrient homeostasis^16,17^. Beside the identification of several drought-regulated miRNAs in rice, the functional implication of miRNA-mediated gene regulation was still not clear. Over-expression of osa-miR393 in rice leads to early flowering, increased tiller number, auxin hyposensitivity and reduced tolerance to salt and drought^18^. The heat stress transcription factor (TF):miR398:Cu/Zn SOD module is critical for heat stress tolerance in *Arabidopsis* ^19^. In rice, while miR164:NAC module plays important role in drought tolerance, over-expression of miR169 leads to enhance drought tolerance in tomato^20,21^. Similarly, miR319 acts as a positive regulator of cold stress tolerance in rice^22^. MiRNAs have also been shown to be involved in grain yield. Over-expression of miR397 increases rice yield by enhancing grain size and panicle branching^23^. Recent report suggests the critical role of miR529a against oxidative stress in rice^24^. Over-expression of osa-miR528 in bentgrass leads to the enhanced tolerance to salt stress and nitrogen starvation^25^.

In recent years, reactive oxygen species (ROS) has emerged as a critical signaling molecule regulating plant drought tolerance via regulation of stomata opening^26–28^. Regulation of ROS homeostasis is critical because controlled ROS generation is important for signaling whereas, excess is deleterious to the system. The redox active micronutrient copper (Cu) plays an important role in ROS homeostasis due to its requirement in both ROS producing (e.g. electron transport) and scavenging mechanisms^29,30^. In addition, the miRNA-mediated regulation of plant Cu proteins is conserved among plants^31^.

In the current study, by a molecular comparative analysis of drought tolerant, Nagina 22 (N22)^32–35^ and sensitive rice cultivar, Pusa Basmati 1 (PB1)^36^ we could delineate a multi-dimensional regulatory network wherein the miRNA nodes function as a junction associating rice copper and ROS homeostasis with drought tolerance in tolerant rice cultivar. This naturally occurring schema involves the interplay of Cu-transporters, TF, miRNAs, Cu-requiring proteins and metabolic enzymes. Drought leads to the lowering of internal Cu levels in the tolerant cultivars (but not in sensitive one). This drought mediated copper deficiency up-regulates cultivar-specific drought responsive (CSDR)-miRNAs (miR408-3p, miR528-5p, miR398b, miR397a, miR1871, miR159f and miR2878-5p) via TF *OsSPL9* [SQUAMOSA PROMOTER BINDING PROTEINS (SBP) like], which in turn down-regulates several genes coding for Cu-containing proteins (e.g. plantacyanins, laccases, Cu/Zn SODs etc.). This ultimately leads to ROS accumulation and enhanced stomata closure in the tolerant cultivar.

## Results

### Identification of Drought responsive miRNAs

Several rice cultivars have a natural capacity to tolerate drought stress conditions and thus their biology is of immense source of knowledge to understand the evolution of molecular nature of tolerance. With this view, we initiated a comparative analysis of the variations in the miRNome of drought-tolerant and -sensitive rice cultivars under similar field drought conditions (Supplementary Fig. S1). Consequently, the drought-induced miRNome of flag leaf and spikelet of the drought-tolerant rice cultivar, Nagina 22 (N22) was compared to that of drought-sensitive cultivar *viz*. Pusa Basmati1 (PB1) at the heading stage of development. Further, selected drought regulated miRNAs were also validated in another set of contrasting rice cultivars i.e. Vandana^35^ (tolerant) and IR64^37^ (sensitive). Drought conditions were simulated in field grown plants of all the four cultivars (see ‘Methods’). Tissue was collected from plants showing the appropriate developmental stage. At molecular level, the extent of drought was confirmed by expression profiling of marker genes *viz*. OsbZIP23 and Rubisco small subunit (RBCS) in flag leaves and OsbZIP23 in spikelets of both the cultivars (Supplementary Fig. S2). Drought is reported to reduce the levels of RBCS^38^ but increase OsbZIP23 (LOC_Os02g52780) transcript abundance^39^. In flag leaf of both the cultivars, drought leads to the significant down-regulation of RBCS while OsbZIP23 was up-regulated (Supplementary Fig. S2), confirming the extent of drought experienced by N22 and PB1 plants. The small RNA from flag leaf and spikelet of two independent biological plants were pooled for control as well as drought conditions. The small-RNA libraries from the flag leaves and spikelets of N22 and PB1 were deep sequenced using illumina GAII platform. All the datasets were analyzed to obtain the expression values as transcript per million (TPM) for all annotated miRNAs as per the miRBase *version* 21 following the pipeline described in methods. As a result, 309, 398, 324 and 438 miRNA candidates were identified in flag leaf and spikelet (both control and stressed) of N22 and PB1, respectively. The overview of deep-sequencing library analysis is listed in Supplementary Table S1.

**Figure 1:**
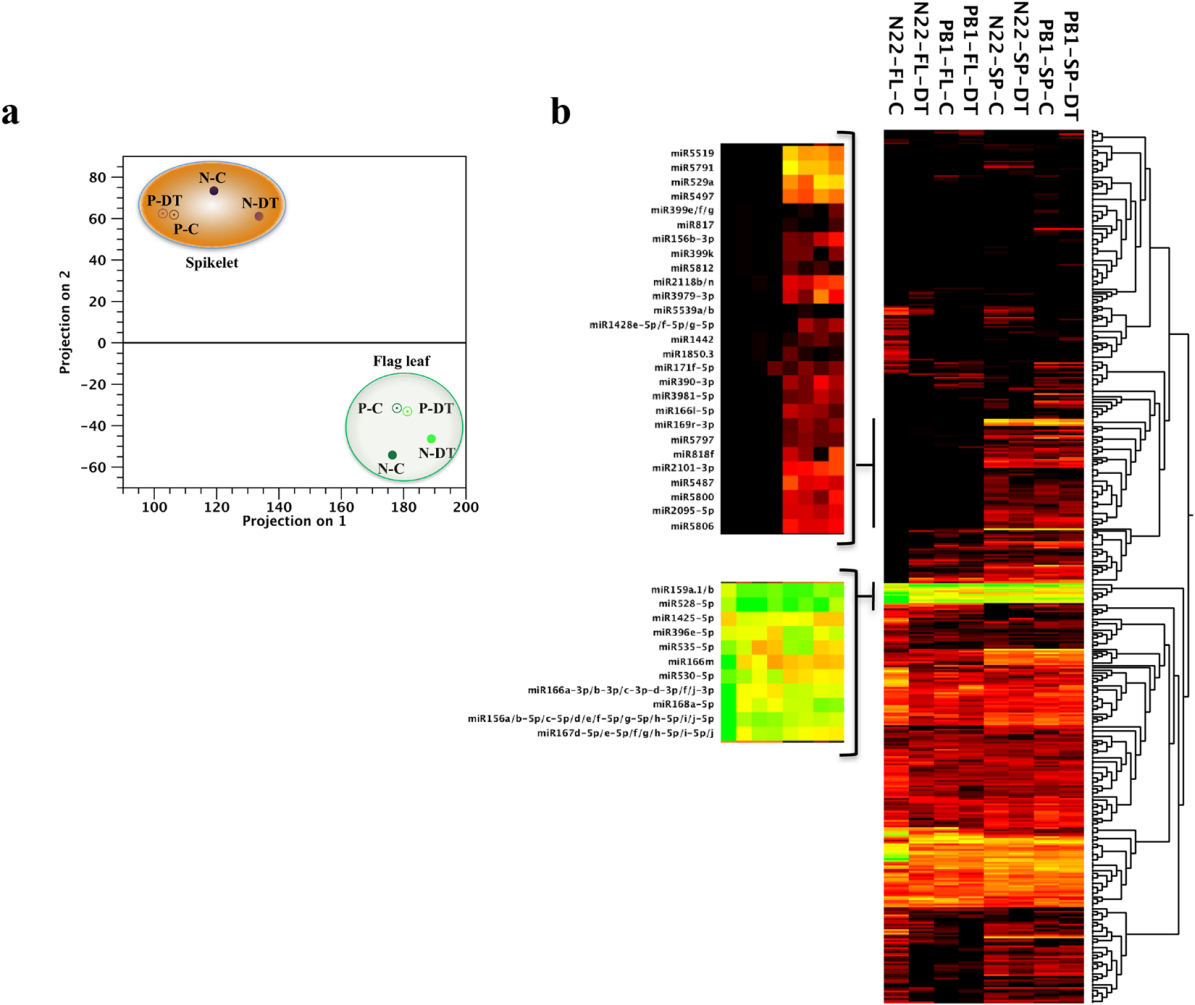
Drought-mediated modulation of N22 and PB1 miRNome. (**a**) Principal component analysis of flag leaf and spikelet miRNome of N22 (N) and PB1 (P) under control (C) and drought (DT) stress conditions. (**b**) Hierarchical clustering of 480 miRNAs depicting the tissue preferential and drought stress regulated miRNA profiles in N22 and PB1 datasets. The clustering was plotted using the log_2_ TPM values following the average linkage parameters. The inset shows the spikelet preferential and highly expressed miRNA clusters.

The miRNome datasets were analyzed using the principal component analysis (PCA) to understand the drought/cultivar/tissue-biased distinction of miRNA expression patterns in contrasting cultivars (Fig. 1a). PCA clearly separates the flag leaf and spikelet datasets into two groups. The data was further analyzed in detail to identify the tissue- and stress-responsive miRNAs. After removing the undetected/low expressing miRNAs (TPM < 10 in all libraries), 208 unique miRNAs were analyzed further. Hierarchical clustering highlights several clusters of miRNAs having tissue biased and/or stress regulated expression patterns (Fig. 1b). Interestingly, a cluster of 11 miRNAs (miR159a.1/b, miR528-5p, miR1425-5p, miR396e-5p, miR535-5p, miR166m, miR530-5p, miR166a-3p/b-3p/c-3p/d-3p/f/j-3p, miR168a-5p, miR156a/b-5p/c-5p/d/e/f-5p/g-5p/h-5p/i/j-5p and miR167d-5p/e-5p/f/g/h-5p/i-5p/j) was highly expressed in all tissue and stress conditions (Fig. 1b). In terms of tissue preferential expression, several miRNAs showed distinct patterns between N22 and PB1. In N22, 23 miRNAs were preferentially expressed (≥20 fold) in flag leaf while only five showed flag leaf enrichment in PB1. MicroRNAs miR408-5p, miR2873a, miR1432-5p, miR1320-5p and miR397b were highly enriched in flag leaf of both cultivars (Supplementary Fig. S3a). Further, in spikelets, 13 and 23 miRNA were preferentially expressed in N22 and PB1, respectively. Six miRNAs having the spikelet preferential expression in N22 and PB1 were validated through qRT-PCR as well (Supplementary Fig. S3b).

**Figure 2:**
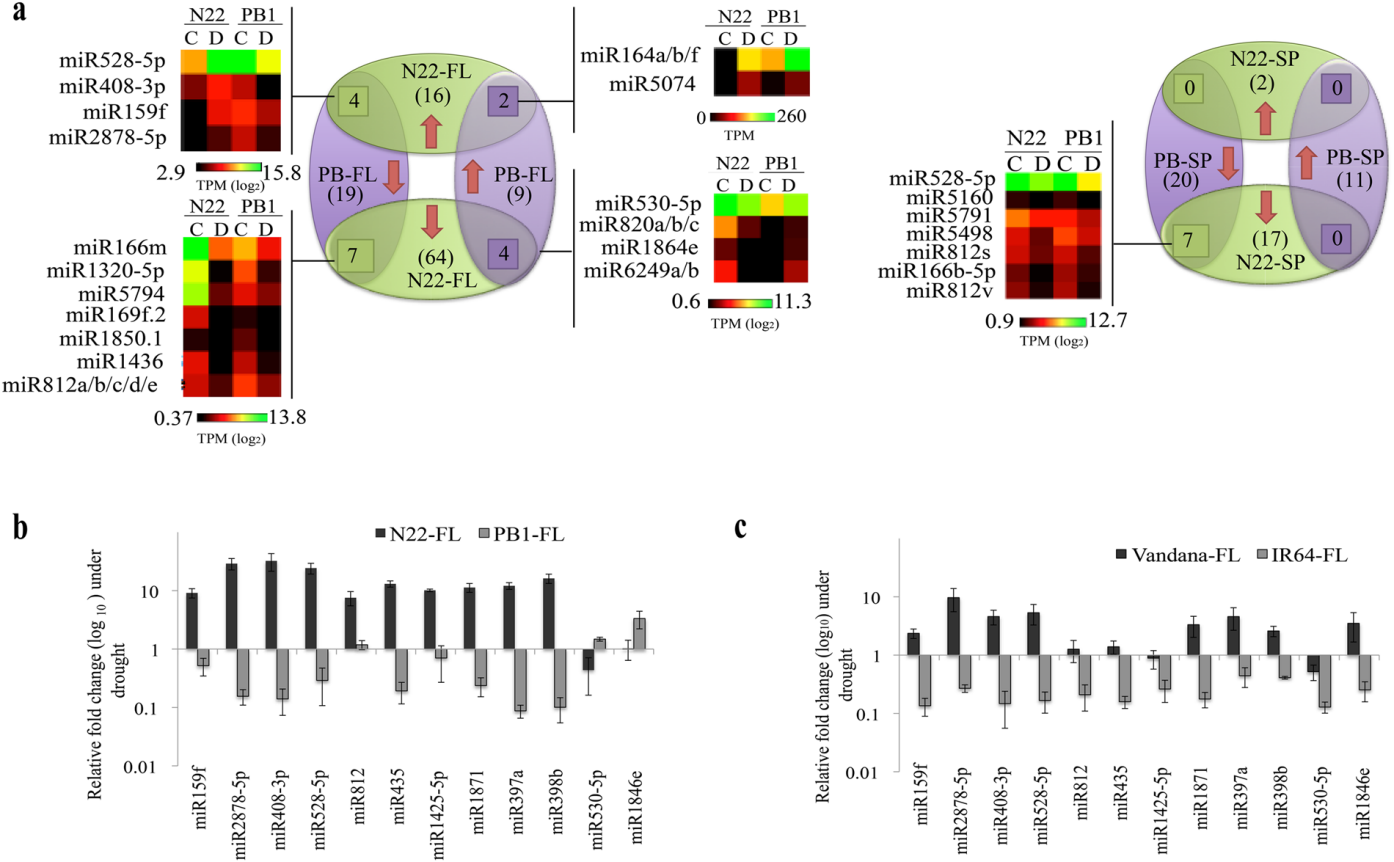
Identification and validation of cultivar-specific drought responsive (CSDR) miRNAs in contrasting rice cultivars. (**a**) Comparison of drought response of miRNAs between N22 and PB1 in flag leaf (FL) and spikelet (SP) in the form of venn diagrams between control (C) and drought (D) datasets. The heat maps were plotted on the log_2_ TPM (Transcript per million) values. (**b–c**) qRT-PCR confirmation of CSDR-miRNAs in flag leaf of N22 and PB1 (**b**) and Vandana and IR64 (**c**) under drought stress at heading stage. 5 S was used to normalize the miRNA transcript levels. Bars represent the standard error. Each expression value represents the mean value of six biological replicates with three technical replicates.

Drought conditions substantially effected the expression of miRNAs in both the cultivars. A total of 80 miRNAs (16/64 up/down-regulated) in flag leaf and 19 miRNAs (2/17 up/down-regulated) in spikelet of N22 were differentially regulated under drought, while a total of 28 miRNAs (9/19 up/down-regulated) in flag leaf and 31 miRNAs (11/20 up/down-regulated) in spikelets were drought responsive in PB1 (Fig. 2a, Supplementary Fig. 4 and Supplementary table S2). The drought responsive miRNome was influenced by both tissue- and cultivar-specific cues. In N22, miR166h-5p, miR166d-5p, miR166b-5p, miR812v, miR5802, miR5540, miR812 and miR5074 followed similar drought response in flag leaf and spikelet while, miR528-5p (up/down) and miR444b.2/c.2 (down/up) followed inverse expression in the two tissues (Supplementary Fig. S4). Similarly in PB1, miR528-5p, miR408-5p, miR812g/h/i/j, miR408-5p, miR812s, miR6249a/b, miR1846e and miR812k/m showed similar drought response while miR169f.2 was differentially regulated between flag leaf and spikelet (Supplementary Fig. S4 and Supplementary Table S2).

A group of miRNAs invoked our interest since they had distinct inverse expression profiles in N22 and PB1. Drought-regulatory miRNAs (fold change in N22/PB1) *viz*., miR159f (15.2/0.35), miR2878-5p (2.66/0.25), miR408-3p (3.94/0.12) and miR528-5p (41/0.08) were significantly up-regulated in N22 flag leaf but considerably down-regulated in PB1 (Fig. 2a). Similarly, miR530-5p (0.38/2.83), miR1846e (0.2/2.48), miR820a/b/c (0.11/2.32) and miR6249a/b (0.02/33.07) were down-regulated in N22 flag leaf but up-regulated in PB1 (Fig. 2a). These inverse expression profiles along with few other miRNAs that showed up-regulation in N22 and slight down-regulation in PB1 (miR812, miR435, miR1425, miR1871, miR397a and miR398b), were confirmed by qRT-PCR in flag leaves of multiple (~6) biological replicates collected from two independent field drought stress experiments (Fig. 2b). The qRT-PCR confirms the drought-induced up-regulation of miR159f, miR2878-5p, miR408-3p, miR528-5p, miR812, miR435, miR1425-5p, miR1871, miR397a, and miR398b in N22 flag leaf. While miR2878-5p, miR408-3p, miR528-5p, miR1871, miR397a, and miR398b were significantly down-regulated in drought sensitive PB1 flag leaves (Fig. 2b). To further assess the nature of this differential expression of miRNAs in drought-tolerant and drought-sensitive cultivars, we extended the study to another pair of drought-tolerant (Vandana) and -sensitive (IR64) cultivars that were also grown under similar growth conditions as N22 and PB1 (in the same field). We found similar expression patterns for seven miRNAs (miR159f, miR1871, miR398b, miR408-3p, miR2878-5p, miR528-5p and miR397a), in the tolerant cultivars, Vandana and N22 (up-regulation) and sensitive cultivars, IR64 and PB1 (down-regulation) (Fig. 2c). As miR159f belongs to MIR159 family, wherein all mature sequence show sequence similarity and target common transcripts, we checked the expression of its other members. Among MIR159 family, three members i.e. miR159f, miR159a.2 and miR159a.1/b are expressed in all datasets and most importantly follow similar expression pattern as miR159f (Supplementary Table S2). Thus, the elevated level of the above seven miRNAs in flag leaf appears to be characteristic of drought tolerant rice cultivars analyzed in the current study (Fig. 2b,c). To further strengthen the nature of cultivar biased differential expression of the above miRNAs under drought and rule out the possibility of a mere outcome of genotypic difference, we performed the two-way ANOVA analysis following the univariate command line with default parameters (IBM SPSS software) for each miRNA expression across all four cultivars (N22, Vandana, PB1 and IR64) and environments (control and drought). For all the miRNAs, there was highly significant interaction (p ≤ 0.001) between the genotype and environment suggesting the contrast in the expression of the above cluster of miRNAs was result of drought-mediated differential response of tolerant vs sensitive cultivar (Supplementary Table S3). As these miRNAs have a cultivar-specific drought response, we referred this group as CSDR-miRNAs (Cultivar Specific Drought Responsive -miRNAs).

### CSDR-miRNAs are majorly involved in the post-transcriptional regulation of copper-requiring proteins

The targets of all drought-regulated miRNAs were identified from the published PARE datasets from our previous study^40^ (Supplementary Table S4). The Gene Ontology (GO) enrichment analysis for all targets was performed using EXPath^41^ (http://expath.itps.ncku.edu.tw/enrichment/rice/enrichment_analysis.php) and the enriched GO category with p-value ≤ 0.01 were considered (Supplementary Fig. S5–9). The results revealed the enrichment of several biological processes and molecular function terms (Supplementary Fig. S5–9). In N22 flag leaf, several stress-related GO terms (Response to stress, response to cadmium ion, response to cold and response to salt stress etc.) in addition to transcription were associated with the transcripts targeted by the up/drought-regulated miRNAs (Supplementary Fig. S5,6). The enriched molecular function (MF) category terms associated with copper ion binding, electron carrier activity, RNA binding, lipid binding and structural constituent of ribosome were in N22 flag leaf (Supplementary Fig. S5,6). In PB1 flag leaf, regulation of transcription (BP), DNA binding and copper ion binding (MF) were significantly over-represented by the targets cleaved by drought regulated miRNAs (Supplementary Fig. S7). Cellular nitrogen compound metabolic process and small molecule metabolic process was enriched in N22 spikelets (Supplementary Fig. S8). While, small molecule metabolic process, translation, response to cadmium ion and copper ion binding was over-represented in PB1 spikelets (Supplementary Fig. S9).

**Figure 3:**
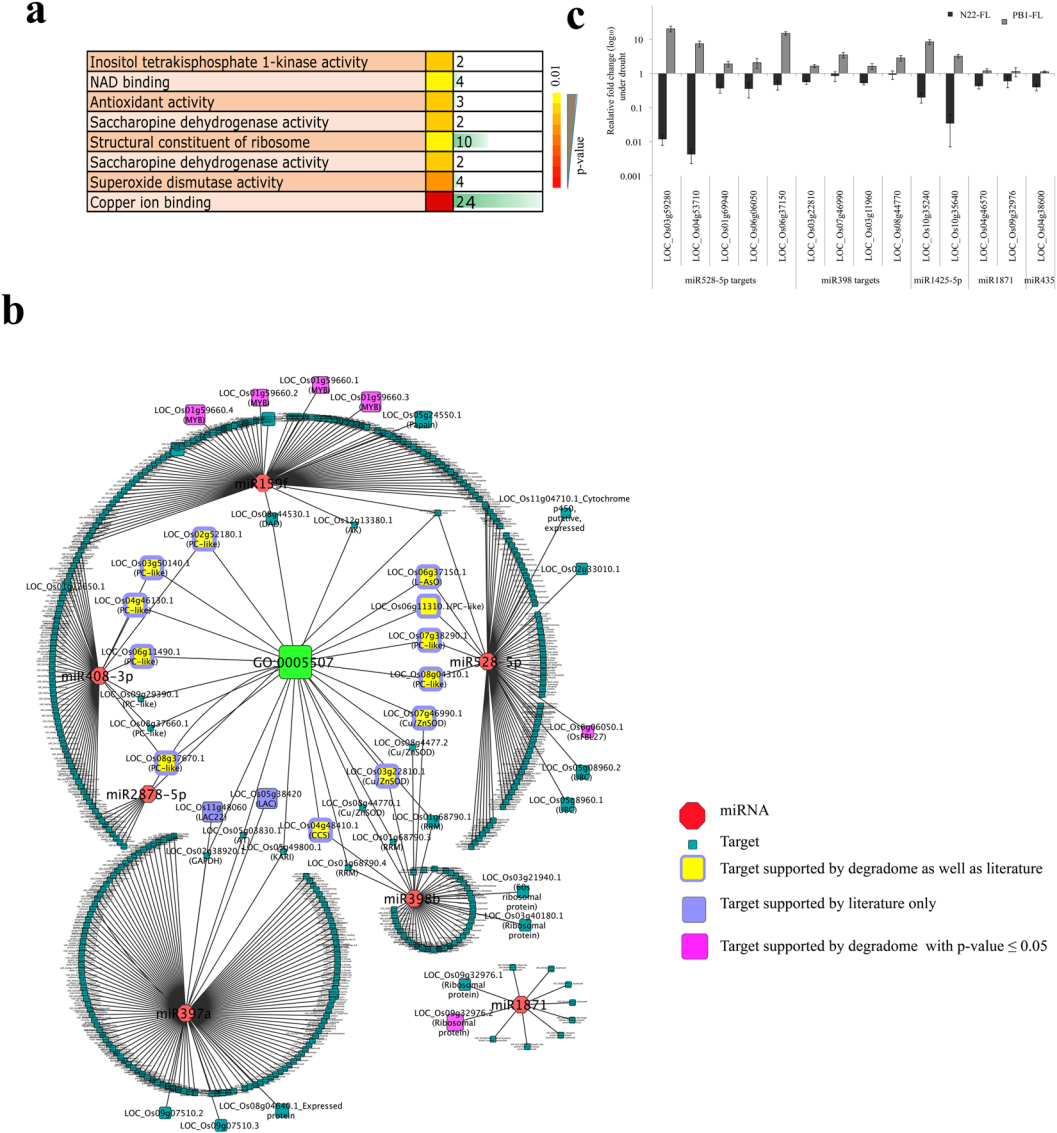
Identification and validation of CSDR-miRNA targets. (**a**) Molecular function enrichment analysis showing the enrichment of “GO:005507-Copper-ion binding” among the CSDR-miRNA targeted 402 genes. Targets were identified through degradome as well as literature. (**b**) CSDR-miRNA:target interaction map showing the association of most valid targets with GO:005507 (Copper ion binding) term. The target node size corresponds to degradome read number (smallest node ≤ 10 reads and biggest node with ≥200 reads). (**c**) Expression analysis of genes targeted by miR528-5p, miR398b, miR1425-5p, miR1871 and miR435. For each real-time expression analysis, at least three biological replicates with three technical replicates were analyzed here. The error bars represent the standard error. Actin was used as the endogenous control.

Further detailed analysis of the CSDR-miRNA:target module identified 402 target transcripts with a cleavage event supported by the PARE data. Functional categorization of these target transcripts showed the enrichment for “Copper ion binding” activity (with a p-value of 6.30E-11) assigned to 24 genes (Fig. 3a). Thus, except miR1871, all other CSDR-miRNAs are majorly involved in the post-transcriptional regulation of proteins that require copper as a cofactor. These targets include several members of plantacyanins/plastocyanin-like domain containing proteins (targeted by: miR408-3p, miR528-5p and miR2878-5p), laccases (miR397a), L-ascorbate oxidases (miR528-5p) and Cu/Zn SODs (miR398b)^23,42–44^ in addition to few others (Fig. 3b and Supplementary Table S5). MicroRNA miR2878-5p targets plastocyanin-like domain containing protein (LOC_Os08g37670) (Fig. 3b and Supplementary Table S5), which is also cleaved by miR408-3p, however, their cleavage sites, are distinct. MiR2878-5p cleaves at 886^th^ position whereas miR408-3p cleaves at 668^th^ position. To determine whether the cultivar specific regulation of CSDR-miRNA is also reflected in the expression pattern of their target transcripts, qRT-PCR was performed for the target genes under similar drought stress conditions. Indeed, these targets also showed cultivar-specific drought response wherein they were down-regulated in tolerant cultivar but up-regulated in sensitive one (Fig. 3b). The cultivar-specific drought response of miR408-3p targets has already been shown in our previous study^45^.

### CSDR-miRNAs are up-regulated under copper starvation

Since CSDR-miRNAs target copper containing proteins, it is possible that they are themselves regulated by internal copper levels. To delineate the mechanism further, we analyzed the pre-miRNA region and the ~2 kb upstream regulatory region of CSDR-miRNA through multiple sequence alignment in all four cultivars. The precursor sequences were found to be highly conserved among four cultivars except for few SNPs observed in MIR408 (1), MIR398b (2) and MIR397a (6) (Supplementary Fig. S10). However, all SNPs are present outside mature miRNA site. Subsequently, the upstream sequence from the precursor start site (for intergenic miRNAs: MIR408, MIR398b and MIR397a) or the host gene start site (for the intronic miRNAs: MIR1871, MIR159f and MIR2878 as well as the exonic miRNA: MIR528) were retrieved from N22, Vandana, IR64 and PB1 genomes (IRDB, Indica Rice Database, http://www.genomeindia.org/irdb/) and aligned using multiple sequence analysis (Supplementary Fig. S11). All the *cis*-regulatory regions contain several stress responsive elements including the presence of several ‘GTAC’ motif or copper response element (CuREs), which is critical for copper responsiveness^16,46^. The putative promoters of MIR159f, MIR1871, MIR408, MIR528, MIR2878, MIR398b and MIR397a harbor 9, 7, 14, 17, 2, 14, and 9 ‘GTAC’ copper responsive motifs, respectively (Supplementary Fig. S11). The regulatory region of MIR1871 harbors several substitutions that clearly distinguish tolerant (N22 and Vandana) and sensitive (PB1 and IR64) cultivars (Supplementary Fig. S11). The upstream regulatory region of MIR159f was highly conserved in all the four cultivars and had only one substitution i.e. T to C in PB1 (Supplementary Fig. S11). The 2 kb regulatory region of MIR528 shows more conservation in IR64 and Vandana while several substitutions were noticed for N22 and few for PB1 (Supplementary Fig. S11). Two such substitutions (T to G and C to A) lead to additional GTAC motifs in both N22 and one in Vandana.

**Figure 4:**
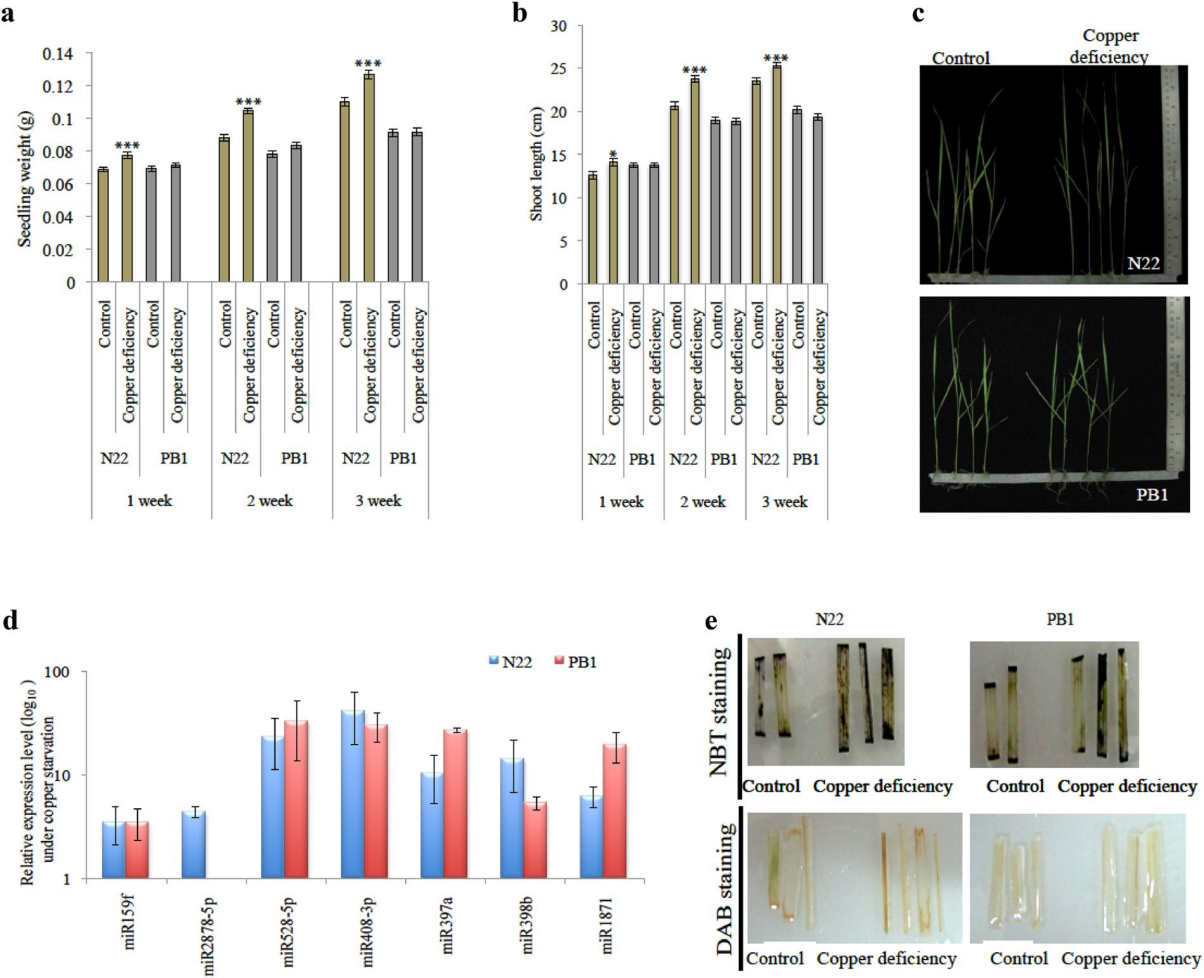
Copper starvation response of rice cultivars. (**a–b**) Comparison of weight and length of N22 and PB1 seedlings under control and copper deficient condition. The error bars represent the standard error (n = 30). The asterisks indicate a significant difference between the indicated samples calculated by two tailed students t-test {P ≤ 0.05 (*), P ≤ 0.005 (**), P ≤ 0.001 (***)}. (**c**) Images of the seedlings grown under control and copper starvation conditions. (**d**) Relative expression profiling of DTA-miRNAs in rice seedlings under copper deficiency administered at seedling stage. 5 S was used to normalize the miRNA transcript level. Five biological replicates were analyzed with three technical repeats. Error bars represent the standard error. The expression of miR2878-5p was not detected in the PB1 seedlings. (**e**) Effect of copper starvation on the accumulation of ROS species (superoxide radical with NBT stain and hydrogen peroxide with DAB stain) in the leaves of N22 and PB1 seedlings.

To investigate the copper response of CSDR-miRNAs, both N22 and PB1 seedlings were challenged with copper starvation conditions. Both N22 and PB1 1-week-old seedlings were transferred to copper +/− rice growth medium in culture tubes. The growth of seedlings was monitored upto 3 weeks of copper starvation. Interestingly, copper starvation induced a visible enhancement in N22 seedling vigor as compared to PB1 (Fig. 4a–c). Expression profiling of CSDR-miRNAs under these conditions revealed that all the CSDR-miRNAs are up-regulated in both the cultivars (drought sensitive and tolerant) at seedling stage (Fig. 4d). Moreover, besides effect on growth and expression of CSDR-miRNAs, copper starvation also led to the generation of ROS in both the cultivars (Fig. 4e).

### CSDR-miRNAs MIR528 and MIR408 are regulated by OsSPL9

**Figure 5:**
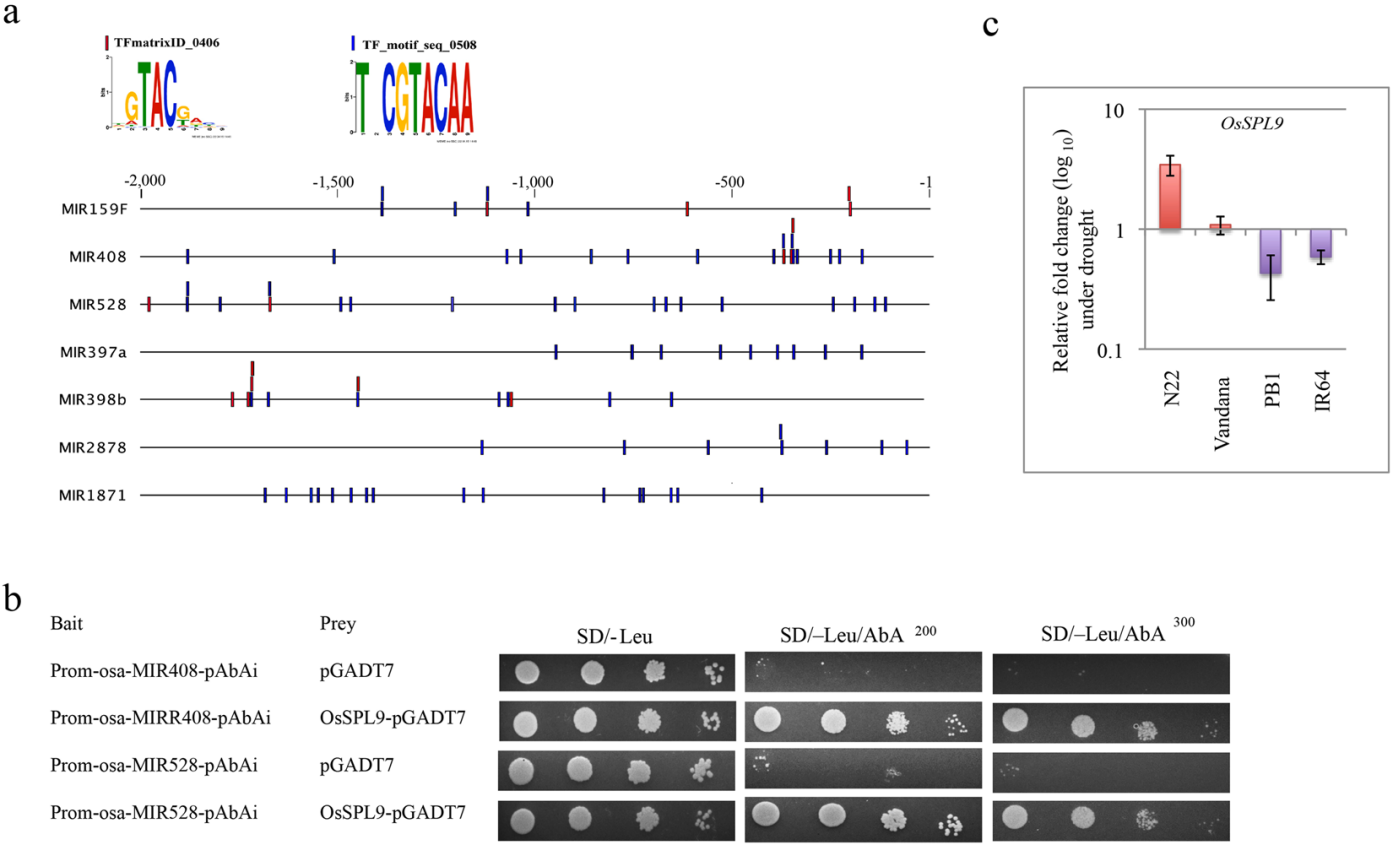
OsSPL9 mediated regulation of CSDR-miRNAs (**a**) The representation of OsSPL9 binding sites on the 2 kb promoter of CSDR-miRNAs. (**b**) Y1H assay of binding of OsSPL9 to the promoter sequence of MIR408 and MIR528. Yeast cells were grown on selective media with different concentrations of 3-amino-1,2,4-trizole (3-AT). (**c**) Real-time expression analysis of OsSPL9 in flag leaf of N22, Vandana, PB1 and IR64 under field drought conditions simulated at heading stage of development. Three biological replicates were analyzed with three technical repeats. Error bars represent the standard error.

In *Arabidopsis*, AtSPL7 (At5g18830) is known to regulate gene expression under copper-limiting conditions by binding to the CuRE elements^47^. On the basis of sequence similarity, OsSPL9 (LOC_Os05g33810) is reported to be the rice ortholog of AtSPL7^48^ and thus may be involved in the regulation of gene expression under copper deficient conditions. As all DTA-miRNA promoters contain the core GTAC motif, we extended the analysis further to look for the OsSPL9 binding sites. Two OsSPL9 binding sites i.e. TFmatrixID_0406 and TF_motif_seq_0508 were reported in the ‘Plant Promoter Analysis Navigator’ (PlantPAN^49^; http://PlantPAN2.itps.ncku.edu.tw) database (Fig. 5a). All CSDR-miRNA contain multiple such OsSPL9 TF binding sites (Fig. 5a). Subsequent analysis based on the basis of yeast-one-hybrid assays, clearly established that the OsSPL9 protein interacts with the 318 bp (Chr1: 12301236-12301553) upstream putative MIR408 promoter containing 11 GTAC elements as well as 196 bp (Chr3: 1667048-1667243) upstream putative MIR528 promoter in rice (Fig. 5b). Interestingly, we also found that the transcript level of *OsSPL9* is up-regulated in N22 and stable in Vandana flag leaves, but are significantly down-regulated in flag leaves of both the sensitive-cultivars, PB1 and IR64 during drought conditions (Fig. 5c). Indeed, promoter of *OsSPL9* gene has many *cis*-acting elements related to drought, salt, ABA and Cu response such as CURECORECR (5), ACGTATERD1 (3), CBFHV (2), MYBCORE (5), and MYCCONSENSUATE (5) (Supplementary Fig. S12). Thus, in rice expression of *OsSPL9* was found to be regulated during drought (Fig. 5c) but its transcript levels was not altered by copper starvation in rice seedlings (Supplementary Fig. S12).

### Flag leaves of drought tolerant cultivars accumulate low copper during drought

**Figure 6:**
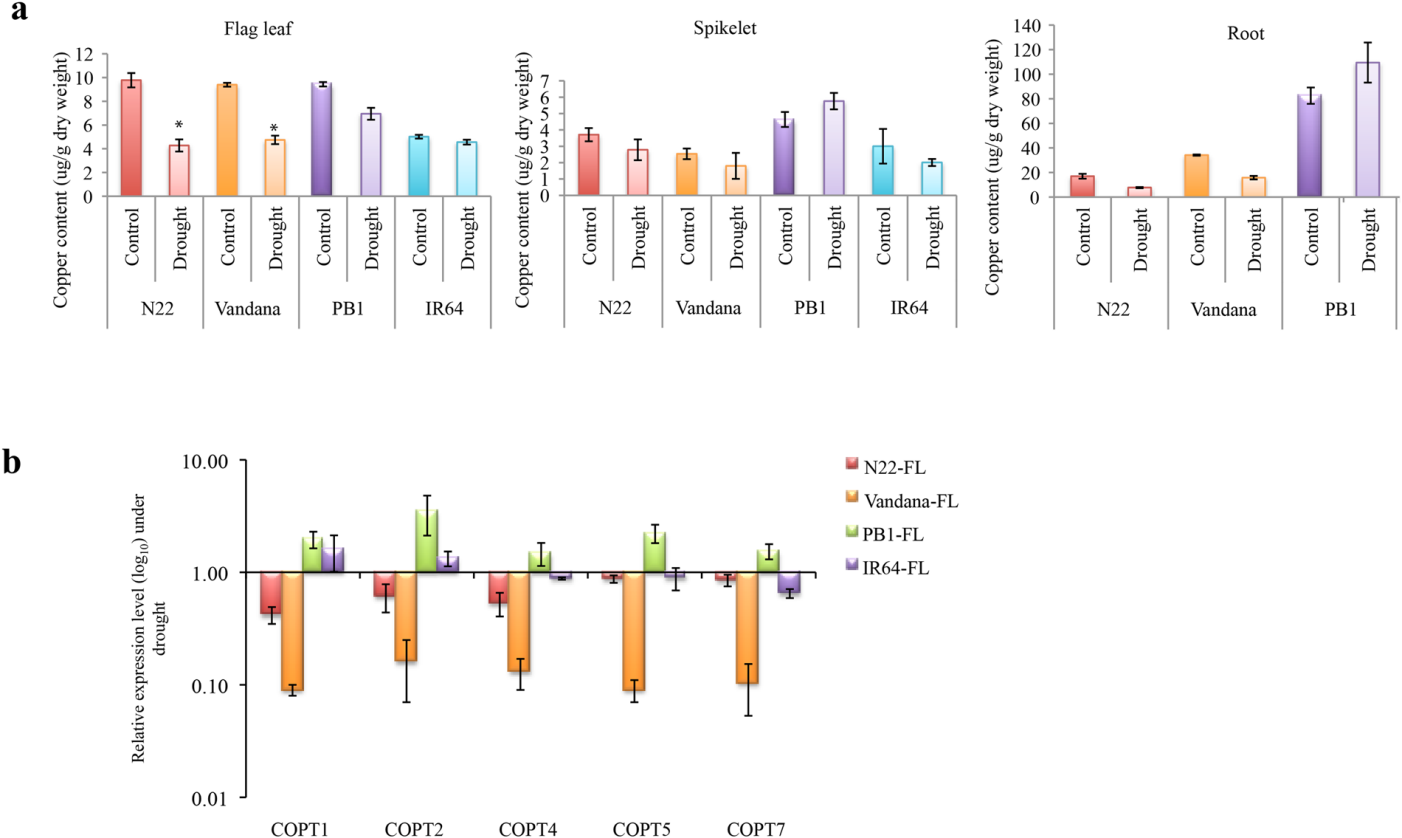
Drought differentially affects the copper homeostasis in drought tolerant and sensitive cultivars. (**a**) Estimation of copper levels in the flag leaf and spikelet collected at heading stage and root collected at milky stage under control and drought stress conditions using atomic adsorption flame spectrophotometer (Soil science, IARI). The graph was plotted with three biological replicates and the bar represents the standard error and the asterisks indicate a significant difference between the indicated samples (two-tailed students t-Test, P ≤ 0.05). (**b**) Expression analysis of genes involved in copper transport was done in the flag leaf of N22, Vandana, IR64 and PB1 at heading stage under drought conditions. At least seven biological replicates and three technical repeats were used for the expression analysis. The error bars represent the standard error. Actin was used as normalization control.

CSDR-miRNAs were found to respond to both drought conditions as well as internal copper levels. Their response to deficient copper levels was similar in the drought-tolerant/sensitive cultivars, but they behave differently during drought. Further, most of them target copper containing protein genes. Incidentally down-regulation of copper requiring proteins can be associated with the copper status of plant^50–52^. Thus, to ascertain whether these two phenomena i.e. response to drought and internal copper levels are independent or inter-related, we analyzed the status of copper levels in the flag leaves, roots and spikelets of the tolerant- and sensitive-cultivars under drought conditions. We found that drought distinctly affects the internal copper levels and that too in a cultivar dependent manner (Fig. 6a). The copper levels in the flag leaves of N22 and Vandana fall significantly as compared to PB1 and IR64 in response to drought (Fig. 6a). The internal copper levels in spikelet do not show any significant variation. In mature roots, the levels are significantly decreased in N22 and Vandana whereas the levels are slightly elevated in PB1 (Fig. 6a).

Further, we assessed the regulation of copper transporters during drought in the tolerant and sensitive cultivars. Indeed, copper transporters *COPT1*, *COPT2* and *COPT4* were significantly down-regulated in N22 and up-regulated in PB1 flag leaf (Fig. 6b). Moreover, copper transporters *COPT5* and *COPT7* were also slightly down-regulated in N22. Further, all copper transporters were down-regulated in Vandana flag leaf as well but remain almost constant in IR64. Additionally, copper transporting ATPases were significantly down-regulated in N22 but not in PB1 flag leaf (Supplementary Fig. S13). Interestingly, the sequence comparison of the promoter regions of rice COPTs in all four cultivars leads to the several tolerant cultivars specific SNPs (Supplementary Fig. S14).

### One major functional impact of CSDR-miRNA activity is reduction of Cu/ZnSOD activity during drought

Fundamentally, there could be multiple functional implications of the unique and cultivar-specific expression behavior of the CSDR-miRNAs. One of the major class of genes targeted by these miRNAs are the plantacyanin genes. Plantacyanins are a multi-gene family coding for copper containing proteins and are relatively less explored^53,54^. Down-regulation of plantacyanins and laccases via miRNAs due to low Cu is known to be a part of the ‘copper economy model’ wherein under low Cu levels several plantacyanins and laccases are down-regulated to re-mobilize Cu for plastocyanin activity^51^. In our analysis as well, we observed a fall in internal copper levels in the flag leaves of the drought tolerant rice cultivars. This could affect plastocyanin levels and consequently the photosynthetic electron transport. To explore this phenomenon we estimated the modulation in the plastocyanin levels under different growth conditions. Under simulated copper starvation in N22 seedlings, plastocyanin transcript levels tend to decreases significantly indicating the dependence of plastocyanin on internal copper levels (Supplementary Fig. S15). However, analysis in the flag leaves wherein the internal copper levels fall during drought conditions (in drought tolerant cultivar), revealed that there is no significant decrease either at the transcript levels or protein levels of plastocyanin (Supplementary Fig. S15).

**Figure 7:**
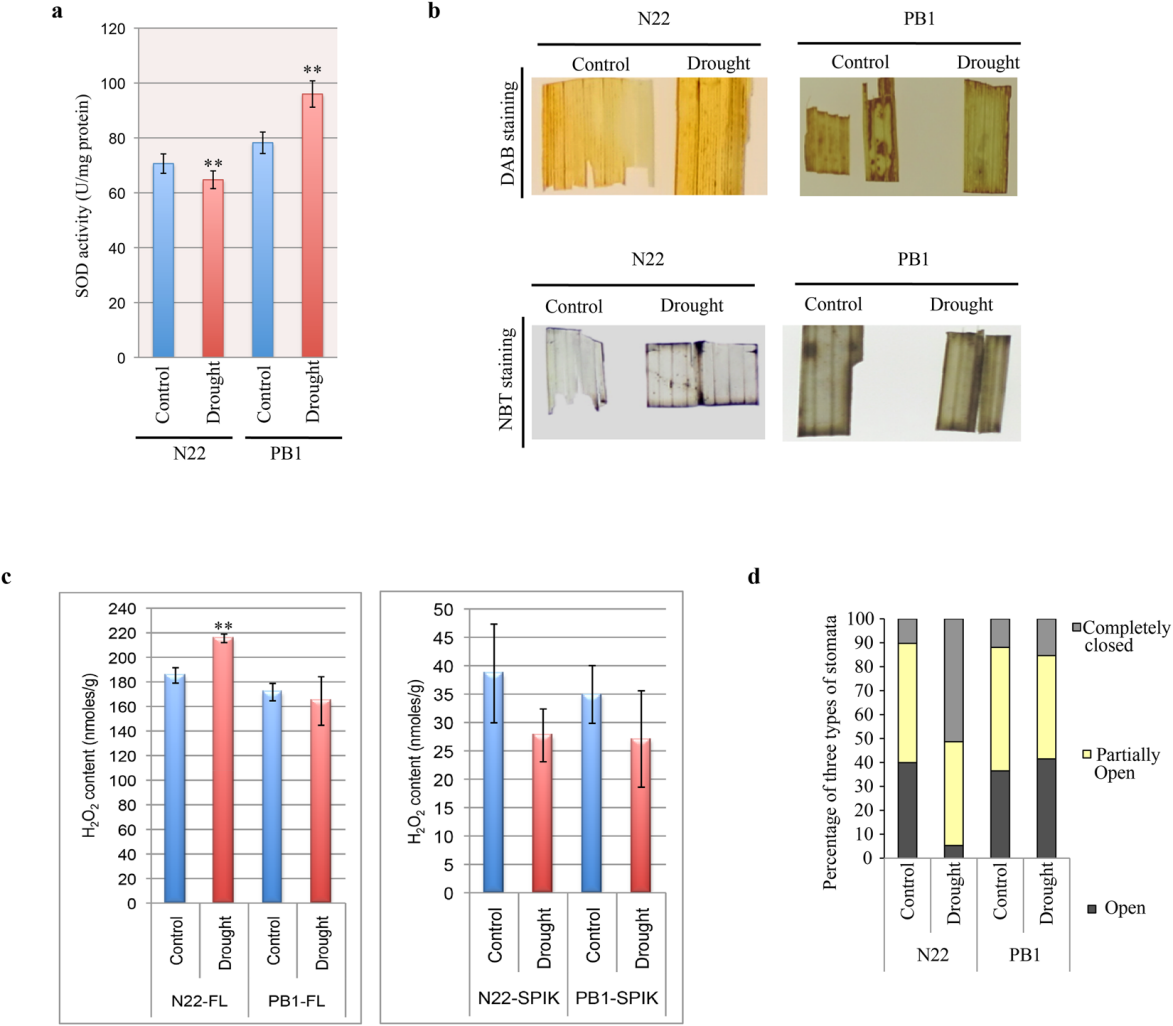
Drought leads to the differential ROS accumulation in rice cultivars. (**a**) SOD activity assay in the flag leaves of N22 and PB1 under control and drought conditions by monitoring the inhibition of the photochemical reduction of NBT. The data represents the mean value of seven biological and three technical replicates each. The asterisks indicate a significant difference between the indicated samples {two-tailed students t-test, P < 0.05 (*), P < 0.005 (**), P < 0.001 (***)}. (**b**) The histochemical staining for detecting the accumulation of H_2_O_2_ by DAB and superoxide ions by NBT in the flag leaf segments of control and drought stressed N22 and PB1. (**c**) The quantitative estimation of H_2_O_2_ in flag leaf (FL) and spikelet (SPIK) of N22 and PB1 under drought stress. The data represents the content on fresh weight basis and eight biological replicates were analyzed for each sample. The asterisks indicate a significant difference between the indicated samples {two-tailed students t-test, P < 0.05 (*), P < 0.005 (**), P < 0.001 (***)}. (**d**) The percentage of three types of stomata (open, partially open and completely closed) in the flag leaves of N22 and PB1 under control and drought stress conditions (n {no. of stomata}; n = 293 for N22 control, n = 265 for N22 drought, n = 277 for PB1 control and n = 287 for PB1 drought).

The second major impact of low copper accumulation and the consequent heightened activity of the CSDR-miRNAs in the flag leaves of the tolerant cultivars under drought would be the reduction in the levels of Cu/ZnSODs. The copper binding Cu/ZnSODs, CCS and L-ascorbate oxidase, targeted by the two CSDR-group members i.e. miR398b and miR528-5p (via glutathione ascorbate cycle) are involved in the removal of superoxide radicals (Supplementary Fig. S16). Thus, both tolerant- and sensitive-cultivars may differ in their ROS homeostasis as well. Our analysis explained in the preceding sections established that the transcript levels of L-ascorbate oxidase and Cu/ZnSODs are significantly decreased during drought in the flag leaves of tolerant cultivars (but not in sensitive cultivars). To further ascertain the miR398-mediated regulation of Cu/ZnSODs, the SOD activity was analyzed in flag leaves of N22 and PB1 under control and drought conditions. As expected the SOD activity was also regulated in an opposite manner in N22 and PB1 under similar drought (Fig. 7a). In N22, there is decrease in the SOD activity in flag leaf consistent with the transcript levels while increase was observed in the flag leaves of PB1 (Fig. 7a).

Further, the cultivar-specific modulation in the SOD activity was also verified by analysis of H_2_O_2_ levels. It could be clearly seen that N22 flag leaf accumulated slightly higher levels of H_2_O_2_ as compared to PB1 under similar drought stress conditions (Fig. 7b,c). Further, other genes (not targets of CSDR-miRNAs) involved in the regulation of ROS homeostasis do not show any significant cultivar-specific regulation (Supplementary Fig. S17). One of the major impacts of elevated H_2_O_2_ levels is the regulation of stomata opening/closing, which is considered a major drought tolerance mechanism^26–28^. Examination of comparative stomata status in the flag leaves clearly showed that in N22, the percentage of completely open stomata decreases from 40 to 5% while the percentage of completely closed stomata increases from 10 to 50% (Fig. 7d and Supplemental Fig. S18) during drought. However, there is only a marginal increase in the number of ‘partially closed’ and closed stomata in PB1 under similar drought conditions (Fig. 7d).

## Discussion

While compelling evidences clearly implicate miRNAs in the regulation of the plant stress response, the understanding is far from complete. Molecular comparative studies on stress tolerant and sensitive varieties help in dissecting the underlying molecular mechanisms and pave a path towards crop improvement^2,55–58^. Thus, the current study systematically compared the miRNome and evaluated the functional impact of the variability in drought tolerant and sensitive rice cultivars during reproductive stage drought regimens. The analysis was initiated primarily on N22 (tolerant) and PB1 (sensitive) rice cultivars and further extended to Vandana (tolerant) and IR64 (sensitive) in order to assess the universality of the findings. The selected rice cultivars provided a good contrast in terms of drought tolerance. Nagina 22 (N22) is an early maturing (90–95 days) aus-type variety with tolerance to drought and heat^32–35^. It has been extensively utilized as a donor in breeding programs for developing heat and drought stress tolerant rice cultivars. Studies have been conducted to identify candidate genes for drought tolerance through comparative transcriptomics^59,60^ and proteomics^61^. Further several QTLs (qDTY1.1 and qDTY3.2) related to grain yield under drought have been mapped to N22^35,62^. Similarly, Vandana is also associated with aus-group as it is developed from aus-type landrace Kalakeri and C22 and contributes qDTY1.1^35^ and qDTY3.2^62^. On the other hand, PB1 is an elite basmati and drought sensitive cultivar while IR64 is a drought sensitive popular lowland cultivar widely grown in India^37^.

The cultivar specific modulation in the miRNome sheds light on the natural variability in the plant response to environmental (drought) cues. It provides evidence for the evolution of unique miRNA mediated regulatory modules in contrasting rice genotypes. In light of the reported studies the present study for the first time gives the comparative aspect of drought regulated miRNome of flag leaf and spikelet at reproductive stage (most sensitive stage to drought) in very crucial rice cultivars. Perhaps the most striking observation was the identification of ‘Cultivar Specific Drought Responsive (CSDR) miRNAs’ that maintain a totally inverse drought mediated expression profiles in the flag leafs and not in spikelet. These included miR528-5p, miR408-3p, miR2878-5p, miR398b, miR397a, miR1871 and miR159f which were significantly up-regulated in the flag leaves of drought tolerant cultivars, N22 and Vandana but down-regulated in sensitive cultivars, PB1 and IR64. These inverse expression profiles had been validated from multiple biological replicates grown at different locations (UDSC and IARI, New Delhi). MicroRNAs miR159f, miR408-3p, miR398b and miR397a are highly conserved miRNA families whereas miR528-5p is monocot specific while miR2878 and miR1871 are specific to rice. As expected, target transcripts of these miRNAs were also found to be modulated in cultivar-biased manner (inversely to that of the miRNAs) as they are down-regulated in N22 and up-regulated in PB1. Our qPCR validation from multiple biological replicates of CSDR-miRNA/target expression patterns in tolerant and sensitive cultivars further confirmed the findings of the deep sequencing data.

While several studies have earlier reported inverse trends in the expression patterns of the miRNome in contrasting cultivars of cowpea under drought^63^, maize under hypoxia^64^ and soybean under nitrogen starvation^57^ and low phosphorus conditions^65^, the uniqueness of this group of miRNAs was that except miR1871, all other CSDR-miRNAs target genes coding for proteins that require copper as a cofactor. miR408-3p targets plantacyanin family members in rice^43–45^, wheat^66^ and *Arabidopsis* ^51,67^ and COX5b in *Medicago* ^68^. Incidentally, a recent study compared and identified the drought-regulated miRNome of Vandana and IR64 in leaf and shoot tissue at vegetative stage^69^ and showed that miR398, miR397 and miR528 follows up-regulation in IR64 and down-regulation in Vandana. However, the qPCR validation in the same study failed to confirm the trends but corresponded to the trends reported in the current study.

Plantacyanins/phytocyanins/Plastocyanin-like-domain containing proteins are copper proteins with an affinity to bind single copper atom and may function in electron transport^53,54,70,71^. *Arabidopsi*s has 38 PCs while rice has 62 OsPCs^54^. miR398 cleaves Cu/Zn SODs in rice, Arabidopsis^16,72^ and common bean^73^, while miR397 is known to target several laccases in rice, *Arabidopsis*, tobacco and populus^23,74^. Recently, it has been shown that the over-expression of miR397 increases rice yield by enhancing grain size and panicle branching^23^. Also in *Arabidopsis* miR397a over-expression improves plant tolerance to chilling and freezing stress^75^. miR397/laccase module act as a master regulator of lignin polymerization and regulate the levels of lignin in *Arabidopsis* ^76^. Apart from the above three, the other two CSDR-miRNAs miR528-5p and miR2878-5p are specific to monocots and also target predominantly copper requiring proteins. While miR528-5p target several plantacyanin members in addition to *L-ascorbate oxidase*, miR2878-5p share same target with miR408-3p (albeit at different sites). Such unique features of the CSDR-miRNA wherein they are drought regulated in a cultivar-specific manner and primarily target Cu-containing proteins suggest a miRNA-mediated interplay of drought response and Cu homeostasis. Indeed, individually, CSDR-miRNAs miR398, miR408 and miR397 have been well explored in plant copper deficiency response wherein all three are induced during copper starvation thereby down-regulating their respective Cu proteins^16,51^. Thus, it is apparent that CSDR-miRNAs while being drought responsive are also involved in copper homeostasis. This observation is further supported by analysis of rice seedlings grown hydroponically under copper limiting conditions. All the CSDR-miRNAs were found to be copper responsive as they were up-regulated under low copper conditions in both drought tolerant and sensitive cultivars. Since all the CSDR-miRNAs target Cu-containing proteins and were regulated by internal copper levels, we compared the cellular copper levels in the plants grown under control and drought conditions. Interestingly, direct measurement of the internal copper clearly indicated that the levels were distinctively different in the flag leaves of control and drought treated rice plants. The modulation was much more evident in the tolerant cultivars than the sensitive ones. The fall in the copper levels is also supported by decrease in the levels of several copper containing proteins (some of which are targeted by CSDR-miRNAs themselves) during drought conditions. Fundamentally, copper is an important micronutrient for plant growth and development. A minimum of 5 µg/g leaf dry biomass is required by plants and greater than 20 µg/g dry biomass can cause copper toxicity. It acts as a cofactor for several proteins such as plastocyanin, cytochrome-c oxidase, Cu/ZnSODs, ascorbate oxidases, amine oxidases, laccases, plantacyanins, polyphenol oxidases and multicopper oxidases^29,77–79^ and plays structural roles in ethylene and salicylic acid receptors^80,81^. Several studies have indicated the role of copper homeostasis in abiotic stress tolerance in Populus^52^ and *Arabidopsis* ^19^ as well as in biotic stress (*Xanthomonas Oryzae*) in rice^82^. Recently, a report provides evidence for the reciprocal cross-talk between Cu status of plant and ABA metabolism and signaling^75^. ABA also plays role in the inhibition of plasma membrane copper transporters and regulates the master regulator AtSPL7 and its targets^83^. Thus, it is interesting to note that internal copper levels are also affected by drought conditions and that too in a cultivar-specific manner. This could be one of the reasons for the cultivar-specific expression of CSDR-miRNAs during drought conditions. At the moment, it is difficult to conclusively ascertain the main reason for lowering of internal copper levels in a cultivar specific manner. Nevertheless, copper transporters may play an important role since they were also found to be differentially regulated (at transcript level) under drought conditions. More studies would be required to completely understand this phenomenon.

Further, the CSDR-miRNAs have several GTAC *cis*-acting motifs in their promoter regions. These motifs are known to be bound by CRR1 or AtSPL7, which is the master regulator of copper homeostasis in *C*. *reinhardtii* and *Arabidopsis*. The transcription of MIR408, miR397 and miR398 is known to be controlled by AtSPL7 and HY5 in *Arabidopsis* ^13,84^. Since OsSPL9 is the rice ortholog of AtSPL7^48^, we confirmed, with the help of yeast-one-hybrid assay, that OsSPL9 regulates the expression of miR408-3p in rice as well. We also confirmed that expression of even miR528-5p is mediated by OsSPL9 in rice. Interestingly, in a manner similar to the CSDR-miRNAs, the expression of OsSPL9 is also differentially regulated during drought in the flag leaf of the tolerant (N22) and sensitive (PB1) *indica* rice cultivars^45^. Similarly, OsSPL9 was down-regulated in IR64 (sensitive), while stable levels were maintained in Vandana (tolerant). Further, similar to AtSPL7^50^, OsSPL9 also has a very mild up-regulation under copper starvation in N22 seedlings. Earlier reports showed that both AtSPL7 and *C*. *reinhardtii* CRR1 expression are independent of the availability of copper in the medium^46,50,85^, suggesting the role of some other interacting partner in mediating the copper deficiency response. One such protein identified is AtKIN17, which physically interacts with AtSPL7 and function in enhancing the copper starvation response and cellular redox balance^47^. Thus, the transcription factor, OsSPL9 apparently plays an important role in regulating the cultivar-specific expression of CSDR-miRNAs.

The consequences of the cultivar-specific and drought regulated expression of the CSDR-miRNA is equally intriguing. Since several target genes are implicated, the impact is expected to be multi-faceted and not simple to comprehend. Nevertheless few aspects stand out. First, plantacyanins appear to play a central role since three CSDR-miRNAs (miR408-3p, miR528-5p and miR2878-5p) target one or the other member of this family. Due to their capacity to bind copper ion, they have been implicated in electron transport. Another relevant phenomenon worth mentioning is the ‘Copper economy model’ which is a major hallmark of the plant copper homeostasis, that operates under low copper condition and is regulated by SPL transcription factor such as SPL7 in *Arabidopsis* ^16^ and CRR1 in *C*. *reinhardtii* ^46^. Under copper limiting conditions, AtSPL7 on one hand regulates the copper transporters COPTs and Cu acquisition proteins like FRO4/5, YSL2 and COPPER CHAPERONE (CCH) to control the copper uptake and distribution in roots^47,85,86^. While on the other hand, it up-regulates miR397, miR398 and miR408 (all identified as CSDR-miRNAs in rice) as well as miR857, which target transcripts of several copper proteins to re-allocate copper for plastocyanin activity^51,85^. Whether such ‘copper economy model’ is operative along with active participation of plantacyanins during drought conditions in tolerant rice cultivars can only be speculated at the moment. This phenomenon is worth a venture since the current study has shown that while there is a fall in the internal copper levels in the flag leaves of tolerant rice cultivars, the levels of plastocyanin remain more or less stable.

Another major impact of the CSDR-miRNAs appears to be on the ROS homeostasis. Indeed, copper homeostasis is closely linked to ROS homeostasis since copper containing proteins are involved in both ROS production (electron transport chain components) as well as scavenging. The current study also showed that both N22 and PB1 seedlings accumulated similar high levels of ROS when subjected to similar Cu starvation, thereby indicating a link between Cu and ROS homeostasis. Under field drought conditions, activity of the CSDR-miRNAs may also affect ROS homeostasis. One of the major reasons would be the up-regulation of the CSDR-miRNA miR398 and the consequent down-regulation of Cu/ZnSOD during drought conditions. The impact on the miR398-Cu/ZnSOD module in the flag leaves of the tolerant cultivar during drought conditions was confirmed both at the transcript as well as the enzymatic activity level. There was a significant decrease in the Cu/ZnSOD activity in the tolerant cultivar vis-à-vis the sensitive ones. It would be logical to assume that this is due to the heightened activity of miR398 (CSDR-miRNA) under drought conditions in the tolerant rice cultivars. Over-expression of miR398 has been associated with increase in H_2_O_2_ levels in tobbaco^87^, rice^88^ and common bean^73^. The cultivar-specific decrease in the Cu/ZnSOD activity is also supported by a corresponding increase in the ROS levels in the tolerant cultivars. Further, other CSDR-miRNAs may also play significant role since they target plantacyanins which would reallocate Cu to seemingly maintain plastocyanin activity and thus the electron transport, which is one of the major ROS producing pathway. Fundamentally, a controlled and subtle increase in ROS levels are reported to play important role in ABA-induced stomatal closure^89^ and thus enhance drought tolerance in rice^24,26^. Indeed, we did observe that there was an enhanced stomatal closure in flag leaves of the tolerant cultivar (N22) as compared to the sensitive one. However, more data is needed in order to conclusively link the two phenomenon.

**Figure 8:**
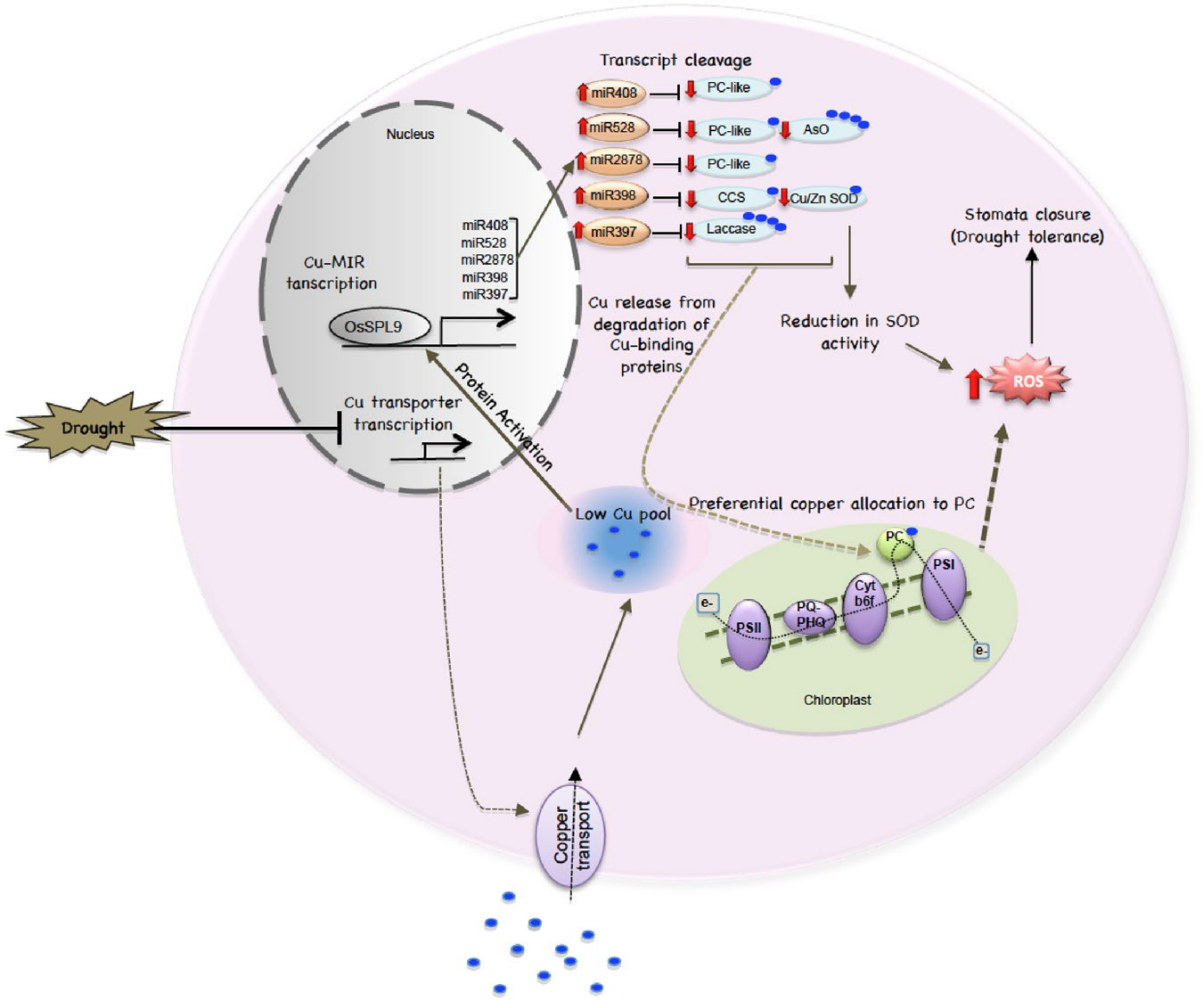
Model summarizing the cross-talk of copper and ROS homeostasis during drought in N22 flag leaf. In brief, during drought there is lowering of internal copper levels in the flag leaves of tolerant cultivar probably due to differential regulation of copper transporters. This leads to up-regulation of *OsSPL9* and consequently of the CSDR-miRNAs. The CSDR-miRNAs then target several copper containing proteins such as plantacyanin and SODs. The observed stabilization of plastocyanin levels even during low cellular copper may indicate the role the ‘copper economy model’ involving the plantacyanins. On the other hand, down regulation of SODs lead to accumulation of ROS. High ROS levels may be responsible for the observed enhanced stomata closure in the tolerant cultivar.

In summary, we depict the major findings of the current study as a model (Fig. 8). The study has revealed several interesting aspects of the interplay of miRNAs and copper-containing proteins, in response to environmental and cultivar specific cues. It establishes a link between copper homeostasis and drought stress response by identifying the evolution of contrasting mode of operation of copper homeostasis network in drought-tolerant and -sensitive rice cultivars. However, whether this phenomenon is specific to the cultivars studied in the current study or a more general phenomenon remains to be investigated. Nevertheless, as per current understanding, the internal copper levels fall in the flag leaf of the tolerant rice cultivars (but not the sensitive ones) in response to drought, thereby affecting the copper homeostasis. A major consequence of the fall in the internal copper levels in the flag leaves of tolerant rice cultivars is the OsSPL9 mediated up-regulation of the CSDR-miRNAs and the concomitant down-regulation of their targets, which would affect two fundamental and inter-related cellular processes viz. copper and ROS homeostasis. Incidentally, copper homeostasis has primarily been studied in response to Cu deficiency/excess in the environment and its active role during other abiotic stress conditions is less explored. The drought-mediated fall in internal copper levels could be due to the drought mediated differential regulation of copper transporters in tolerant and sensitive cultivars. Copper transporters are primarily regulated at the transcriptional level and thus modulation in the transcript abundance during drought would have a significant effect on copper transport, as observed in the study. However, further studies are needed to conclusively implicate the copper transporters. Moreover, the reason for the cultivar-specific transcriptional regulation of transporters also remains to be seen. Thus, the current study has unraveled several interesting facts as well as raised some very relevant questions which are worth a pursue in future.

## Methods

### Plant Material

The seeds of *Oryza sativa* L. *cv* N22, Vandana, PB1 and IR64 were kindly provided by Dr. A.K Singh, Division of Genetics, IARI, New Delhi, Pusa. The rice cultivars were grown in fields and drought stress was simulated at heading stage of panicle development to mimic the natural field drought conditions. As the heading time of each cultivar is different, the experiment was planned in such a way that similar stress was given to each cultivar. To give stress at heading stage, water supply was stopped 10 days before the expected ‘mean heading date’ of each cultivar. Stress was measured by estimating the soil moisture content (below 15% was taken for drought stress) and analyzing the leaf rolling (Supplementary Fig. S1). Portable rain-out shelters were used to avoid rain water. The tissue was collected from plants showing the appropriate developmental stage. Flag leaf (Heading stage) and spikelets (Heading stage) and roots (milky stage) from control and stressed plants were collected and immediately frozen in liquid nitrogen and stored at −80 °C.

For copper starvation experiments, rice seeds were sterilized and maintained under culture room conditions^45^. Seedlings were maintained hydroponically in rice growth medium containing 0.01 ppm copper^90^ for about 1 week and then allowed to grow in rice growth medium without and with copper for 3 weeks.

### Small RNA c-DNA library construction, sequencing and analysis

Small RNA libraries were prepared from flag leaf and spikelet at heading stage of N22 (N22 flag leaf control: SRR2458631; N22 flag leaf drought: SRR2293667; N22 spikelet control: SRR2458943; N22 spikelet drought: SRR2459139) and PB1 from control as well as drought stressed plants. Small RNA cDNA libraries were prepared as per the instructions of Illumina small RNA v1.5 sample preparation kit from 2 µg (1 µg each from two biological replicates) of small RNA. Further, the cDNA was analyzed on MCE^®^-202 MultiNA: Microchip Electrophoresis System for DNA/RNA Analysis (Shimanzu-Biotech)’ for the assessment of quality, size, and concentration and sequenced on the Illumina GAII sequencing platform followed by trimming of adapter using the Small RNA analysis tool of CLC genomics workbench *ver*. 4 with following trim settings: Illumina small RNA adapter (CAAGCAGAAGACGGCATACGA), strand (Minus), action (Discard when not found), score [2, 3,10, 4] and removal of sequencing reads shorter than 15 nt and greater than 35 nt. The trimmed reads were then mapped to N22 and PB1 genome (Indica Rice Database; http://www.genomeindia.org/irdb/) the genome matched reads were retained while the reads matching to the rfam were excluded. The mature (553 unique sequences) and precursor sequences were retrieved from miRBase^91^ (version 21; www.mirbase.org). The genome matched trimmed reads were used as a query against the database of mature miRNA sequences of rice in the Blastn analysis. To identify the miRNAs from N22 and PB1 datasets sequences with a coverage score of  ≥90% of the miRBase mature were further analyzed. All the expression values are normalized as TPM (tags per million = Number of reads * 10^6^/ total number genome matched reads). Further the low abundance miRNAs with a total normalized abundance from all the datasets ≤10 were discarded and the remaining 208 mature sequences were considered for the analysis. Identification of drought responsive miRNAs were done on the following criteria; first the miRNA expression should be ≥10 in any one condition i.e. control or stress. Secondly, the differentially regulated miRNAs should have ≥2 fold up/down regulated (stress/control).

### mRNA and miRNA real time expression analysis

The quantitative expression analysis of miRNAs and mRNAs were performed as per the protocol in our previous study^45^. The expression level of miRNA and mRNA was normalized using 5 S rRNA’s and Actin expression respectively as an endogenous control. ∆∆Ct method was employed to calculate relative fold change (2^−∆∆Ct^) in expression and standard error was calculated. The details of primers used in the study are provided in Supplementary Table S6.

### miRNA target identification and GO enrichment analysis

Targets of known miRNAs were determined using CleaveLand version 3.0 target prediction suit^92^ using the published PARE datasets of rice *viz*. GSM455939 (Inflorescence), GSM455938 (Three week old seedlings), GSM960648 (*O*. *rufipogon*, 4 leaf stage), GSM476257 (Young inflorescence), GSM434596 (Seedling) and PARE libraries prepared from N22 flag leaf and spikelet^40^ at heading and anthesis stage respectively. Cleavage events obtained between 10 and 11 base from the 5′ end of the miRNA were considered with following cutoffs; p-value ≤ 1, number of cleaved reads ≥ 5 (sum from all the datasets).

The Gene Ontology (GO) enrichment analysis was performed using the ExPATH database^41^ and the enrichment terms were visualized using the customized comparison tool of AgriGO (http://bioinfo.cau.edu.cn/agriGO/). The GO terms with ≤0.01 pvalue were considered.

### Genomic organization, precursor and promoter sequence analysis of DTA miRNAs

The precursor sequences of the miRNAs were retrieved from the miRBase version 21 (www.mirbase.org) and searched in the RGAP database (rice.plantbiology.msu.edu) using BLAST tool against the genomic sequences to determine the genes in which they reside. Similar search was also performed against the full-length cDNA clones available at KOME (http://cdna01.dna.affrc.go.jp/cDNA/). Then the precursor and 2 kb of the promoter sequences of all the DTA-miRNAs were also retrieved from the Indica Rice Database (http://www.genomeindia.org/irdb/) for N22, Vandana, PB1 and IR64. Multiple sequence alignment of precursor and promoter sequences from different cultivars was performed using the ‘Create Alignment tool’ of CLC Main workbench 5.6.1. Further the promoter sequences from N22 were analyzed for the presence of cis-regulatory elements using PLACE database (http://www.dna.affrc.go.jp/PLACE/) and the OsSPL9 binding sites were mapped using PlantPAN 2.0^49^.

### Histochemical and quantitative estimation of ROS

The detection of H_2_O_2_ using 3,3′-diaminobenzidine (DAB, Biobasic) and superoxide ions using nitrotetrazoluim blue chloride (NBT, SRL) was performed as per the method described^93^ in flag leaf pieces. The concentration of H_2_O_2_ in flag leaf and spikelets were determined by the Amplex Red Hydrogen peroxide/peroxidase assay kit (Invitrogen) as per the manufacturer instructions. Tissue was ground in liquid nitrogen and 30 mg of powder was used for the assay. The fluorescence (excitation at 560 nm and emission at 590 nm) was measured using Infinite M 200 PRO microplate reader (Tecan).

### Yeast-one-hybrid

Yeast one hybrid assay was performed using Matchmaker Gold Yeast One-Hybrid library screening system (Clonetech) as per the instructions. 318 bp (using primers; pro-408-318-F: 5′-CTCGAGCGTCTCGATGAGATGTACGAC-3′ and pro-408-318-R: 5′-GAGCTCCACACGCAACGCAATGTCTTTG-3′) and 196 bp (using primers; pro-528-196-F: 5′-CTCGAGCGTCGCCGTTAGAGCGTTTGCT-3′ and pro-528-196-R: 5′-GAGCTCCCATTGGTGAAGGGTGGCATATT-3′) of region containing the GTAC motif upstream of the MIR408 and MIR528 precursor, respectively, was PCR amplified from the genomic DNA from N22 flag leaf and cloned into pAbAi vector, harbouring the AUR1-C gene. The plasmid was linearized and integrated into the Y1H gold yeast genome. Full-length ORF of OsSPL9 was PCR amplified (SPL9-orf-F: 5′-GAATTCATGGACGCCCCCGGCG-3′ and spl9-orf-R: 5′-CCCGGGCTATGATGAGTAGTTCCTAGACAA-3′) from the cDNA prepared from N22 flag leaf and transformed into the pGADT7-AD vector. All the clones were confirmed with digestion and sequencing. The interaction was determined based on the ability of co-transformed yeast cells to grow on minus Leu medium with 200–300 ng/ml Aureobasidin A.

### Copper content determination

For the copper content analysis, flag leaf, spikelet, and root were harvested, dried at 80 °C for 3–4 days and processed to make in powder. 100 mg of each sample was digested with 4 ml of nitric acid for 2 days and heated to evaporate the acid till the fumes stops. After cooling 2 ml of hydrogen perchloric acid was added and again heated till small volume remained. Then it was diluted to 10 ml with double distilled water, filtered and analyzed using atomic adsorption spectrometry (Soil science, IARI).

### Western blot analysis

The primary antibody against plastocyanin was obtained from agrisera (www.agrisera.com). Total protein was extracted from the flag leaf at heading stage and shoot of three week old plants using the reagents of P-PER^®^ Plant Protein Extraction Kit (Thermo Scientific) after homogenizing 30 mg tissue in liquid nitrogen in motor and pestle. Ten µg of protein was subjected to the Tricene-SDS-PAGE (18%) for separation and transferred to nitrocellulose membrane by electro-blotting. The blots were developed using the Pierce^®^ Fast Western Blot Kit SuperSignal^®^ west femto substrate (Thermo Scientific) following the instructions specified by the manufacturer.

### Measurement of superoxide dismutase activity

The superoxide dismutase activity was measured by the NBT (Nitroblue tetrazolium reagent) reduction method as reported^94^.

### Rice stomata imaging

The flag leaves of control and drought stressed N22 and PB1 plants were analyzed for the percentage distribution of open, partially open and completely closed stomata as per the method^26^ using Zeiss EVO 40 scanning electron microscope.

### Statistical Analysis

Two-way analysis of variance (ANOVA) using the Univariance command line of IBM SPSS statistics (trial version) was to assess the statistical interaction between different genotypes and environments (control and drought) in determining the CSDR-miRNAs expression in contrasting cultivars under drought. The analysis was performed on the dCt values (obtained from q PCR in three biological repeats) of miRNAs under control and drought conditions for each cultivar. The p ≤ 0.05 was considered as significant. A students t-test was used to assess the statistical significance of other quantification data.

### Data availability statement

The sources of small RNA and degradome datasets used in the present study are listed in the Supplementary Table S7. The datasets that are not publicly available are available from the corresponding author on reasonable request.

## Electronic supplementary material

Supplementary Figures

dataset S1

Dataset S2

Dataset S3

Dataset S4

Dataset S5

Dataset S6

Dataset S7
