# Supplementary material for: Identification of miRNA-mediated drought responsive multi-tiered regulatory network in drought tolerant rice, Nagina 22
Source: Sci Rep. 2017 Nov 13;7:15446. doi: 10.1038/s41598-017-15450-1 (PMC5684420; doi:10.1038/s41598-017-15450-1)
Supplement: Supplementary file 1 — Supplementary Figures [file 41598_2017_15450_MOESM1_ESM.pdf]

Supplementary Information

Identification of miRNA-mediated drought responsive multi-tiered regulatory network in drought tolerant rice, Nagina 22

Sonia Balyan[1], Mukesh Kumar[1], Roseeta Devi Mutum[1], Utkarsh Raghuvanshi[1], Priyanka Agarwal[1], Saloni Mathur[2], Saurabh Raghuvanshi[1*]

Supplementary Information

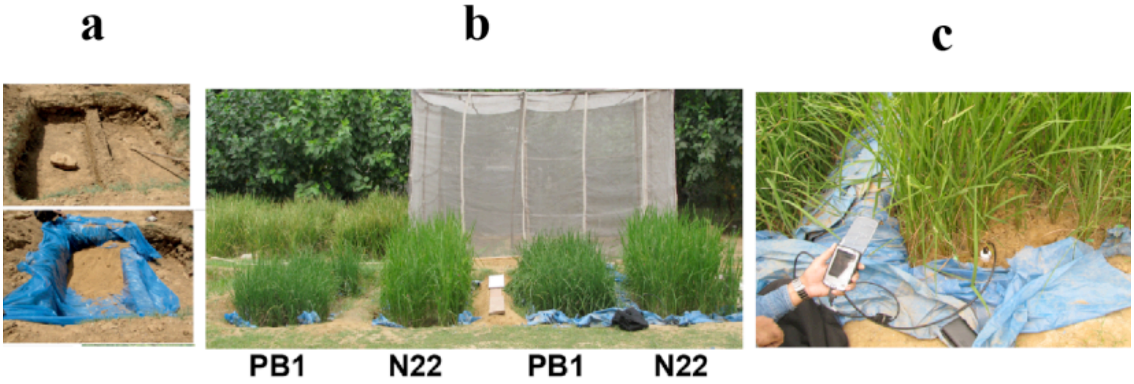

Supplementary Fig. S1. Field set up for implicating drought stress on rice cultivars. (**a**) Three feet deep pits were lined with polyethene were prepared to regulate water supply and avoid the water leakage from outside. (**b**) N22 and PB1 plants growing in the specially designed fields. (**c**) Picture showing the measurment of soil moisture content in the fields.

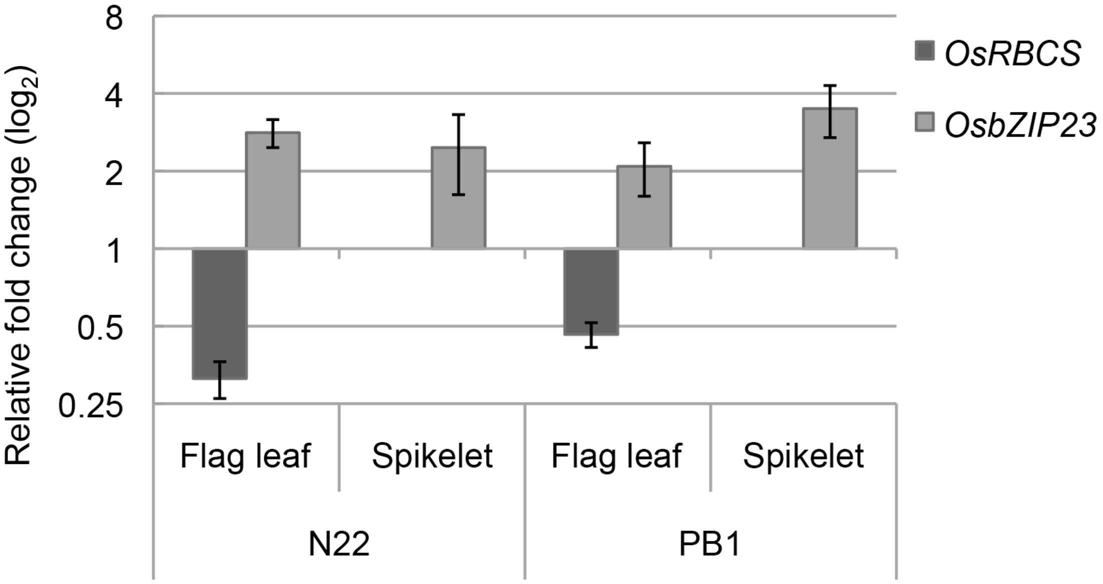

Supplementary Fig. S2. qRT-PCR analysis of Rubisco small sub-unit (*RBCS*) in flag leaf and *OsbZIP23* in flag leaf and spikelet of N22 and PB1 under drought stress at heading stage. Four biological replicate were performed and the standard error is represented as error bar on the top of the bar.

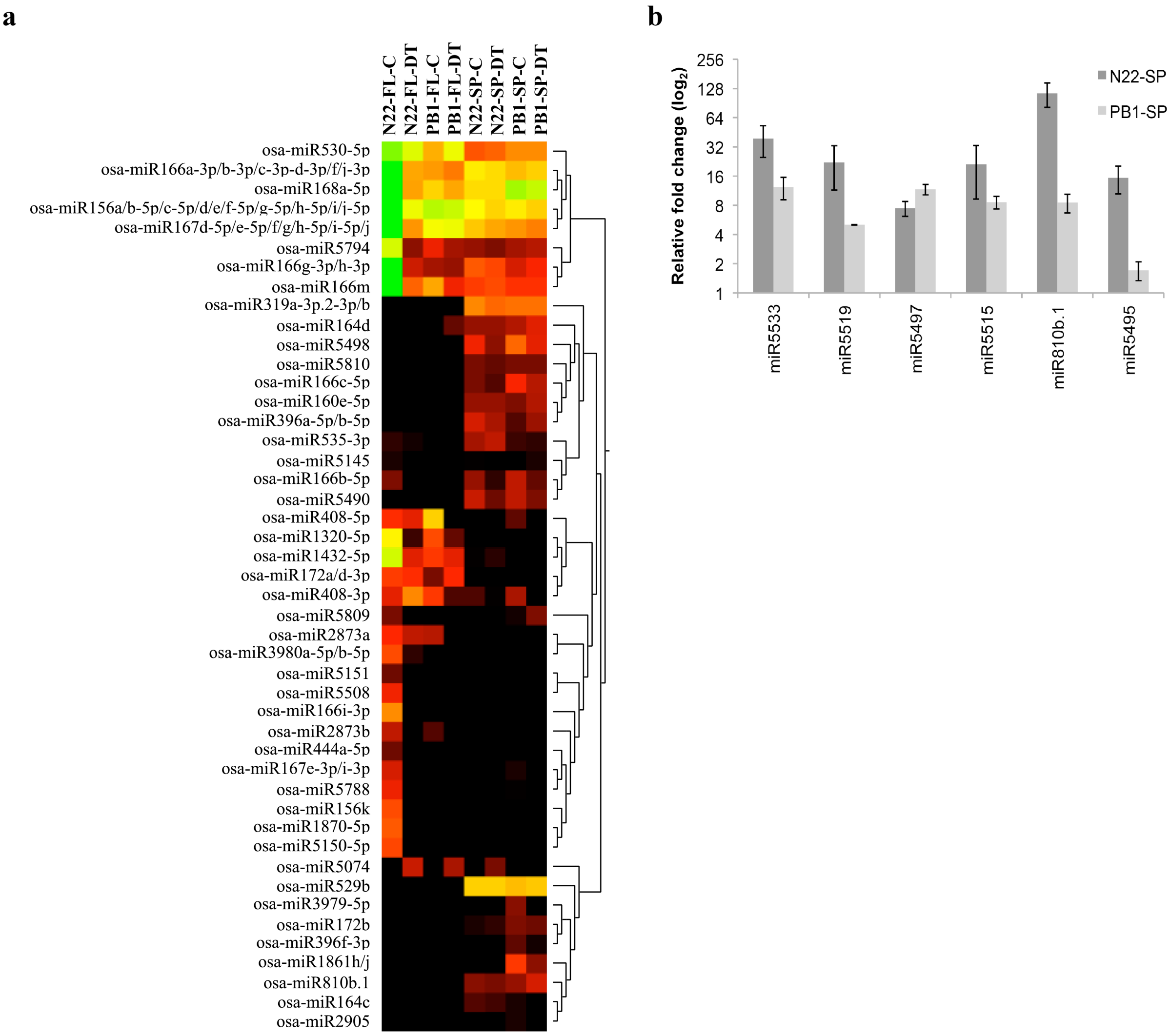

Supplementary Fig. S3. Flag leaf and spikelet preferential miRNAs in N22 and PB1. (a) Hierarchical clustering of log_2 transformed expression levels (TPM) of flag leaf and spikelet enriched miRNAs (having 10 fold higher expression in one tissue w.r.t to the other) in N22 and PB1. The clustering was performed using the Euclidean distance algorithm with average linkage. **(b)** qRT-PCR analysis of spikelets enriched miRNAs in spikelets relative to the expression in flag leaf of N22 and PB1, respectively. The expression represents the data of 3 biological replicates with three technical repeats. The error bar represents the standard error. FL- flag leaf; SP- spikelet; C- control; DT- drought.

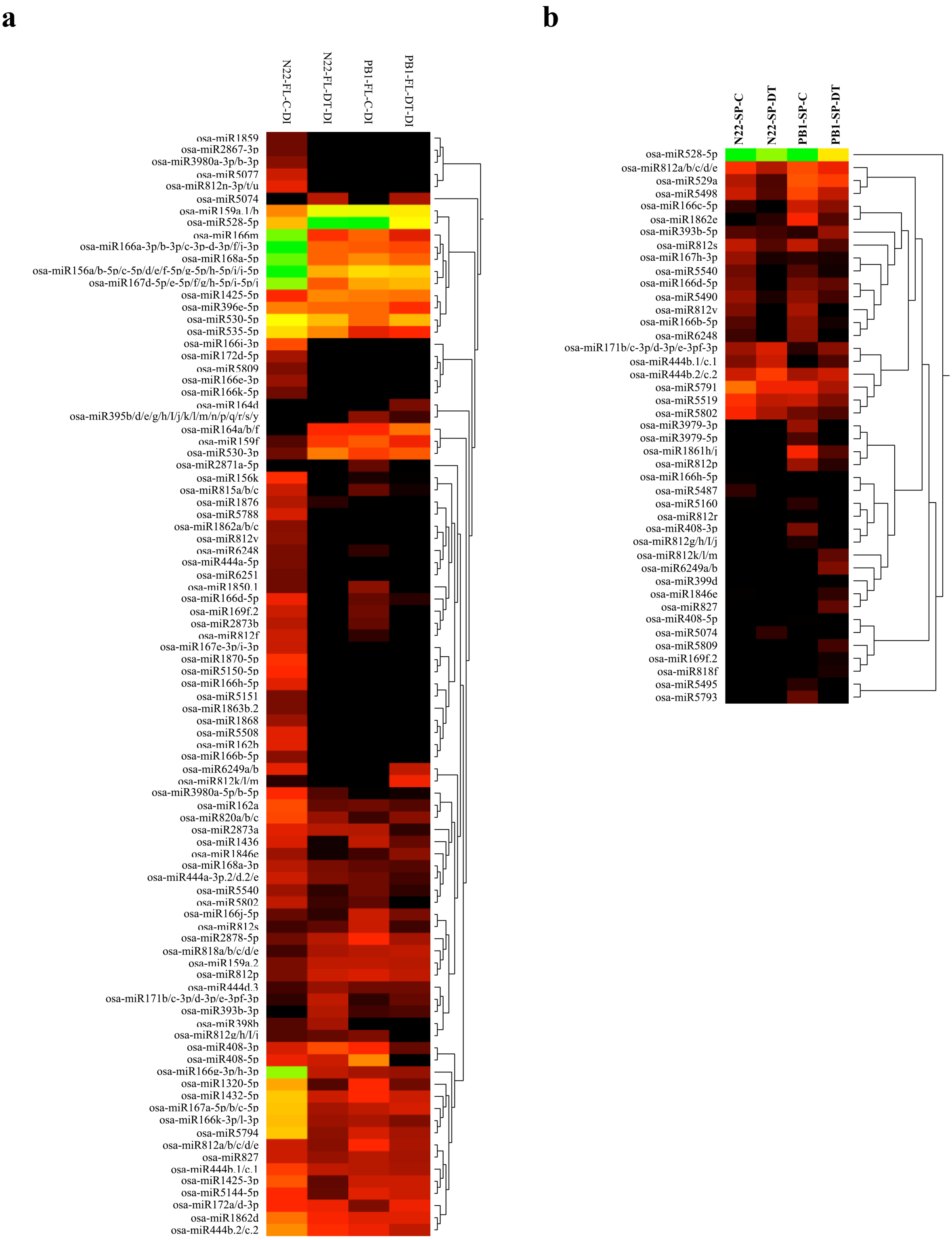

Supplementary Fig. S4. Drought regulated miRNAs in flag leaf and spikelet. Hierarchical clustering of log_2 transformed expression levels (TPM) of drought responsive miRNAs in flag leaf **(a)** and spikelet **(b)** of N22 and PB1. miRNAs with ≥ 2 fold up/down regulation in flag leaf of spikelet dought samples w.r.t. respective control in N22 and PB1. The clustering was performed using the Euclidean distance algorithm with average linkage.

Biological Process

**Adaxial/abaxial axis specification**	2
adaxial/abaxial pattern specification	2
Adventitious root development	3
Anatomical structure development	38
ATP synthesis coupled proton transport	3
Auxin metabolic process	3
Auxin polar transport	4
auxin-activated signaling pathway	9
Biological_process	299
Biosynthetic process	118
Catabolic process	43
Cellular amino acid metabolic process	18
Cellular carbohydrate metabolic process	3
Cellular component assembly	19
Cellular nitrogen compound metabolic process	85
Chlorophyll metabolic process	3
Cinnamic acid biosynthetic process	2
Cofactor metabolic process	13
Defense response to bacterium	10
Determination of bilateral symmetry	5
Electron transport chain	3
embryo development	15
embryo development ending in seed dormancy	11
embryonic pattern specification	4
Endonucleolytic cleavage in ITS1 to separate SSU-rrna from 5.8S rrna and LSU-rrna from tricistronic rrna transcript	2
SSU-rrna from	2
Flower development	8
Gene silencing by mirna	3
Gene silencing by RNA	5
Generation of precursor metabolites and energy	16
glycolytic process	6
Hydrogen peroxide biosynthetic process	3
Innate immune response	3
Integument development	2
Leaf morphogenesis	7
L-phenylalanine catabolic process	2
Macromolecular complex assembly	16
Malate metabolic process	3
Meristem initiation	5
modulation by virus of host morphology or physiology	2
Mrna transport	2
Photorespiration	7
Phyllome development	2
Polarity specification of adaxial/abaxial axis	4
Posttranscriptional gene silencing	3
Primary cell wall biogenesis	2
Primary shoot apical meristem specification	4
Proteasome core complex assembly	4
Protein complex assembly	12
Protein folding	12
Proteolysis involved in cellular protein catabolic process	5
Proton transport	4
Purine nucleotide transport	2
Pyridoxine biosynthetic process	2
Radial pattern formation	2
Regulation of cell cycle process	3
Regulation of lipid metabolic process	2
Regulation of photomorphogenesis	2
response to auxin	12
Response to cadmium ion	35
Response to cold	18
Response to far red light	7
Response to fructose	4
response to hormone	4
Response to light stimulus	10
Response to misfolded protein	5
Response to salt stress	18
Response to stress	58
Response to sucrose	6
Ribosomal small subunit biogenesis	2
RNA interference	4
shoot system development	4
Small molecule metabolic process	51
Spermidine biosynthetic process	2
Sphingolipid biosynthetic process	2
Stomatal lineage progression	3
Symbiosis, encompassing mutualism through parasitism	7
transcription, DNA-templated	31
Translation	27
Translational elongation	5
Tricarboxylic acid cycle	5
Ubiquitin-dependent protein catabolic process	11
Virus induced gene silencing	5
Vitamin B6 biosynthetic process	2

Cellular Component

Apoplast	22
Cell	237
Cell wall	26
Cellular_component	262
Chloroplast	69
Chloroplast envelope	25
Chloroplast stroma	29
Cytoplasm	168
Cytoskeleton	10
Cytosol	60
Cytosolic ribosome	17
Cytosolic small ribosomal subunit	8
Eukaryotic translation elongation factor 1 complex	2
External encapsulating structure	21
Extracellular region	21
Golgi apparatus	19
Intracellular	223
Membrane	75
Mitochondrial proton-transporting ATP synthase complex, catalytic core F(1)	2
Mitochondrial respiratory chain complex I	7
Mitochondrion	38
Nucleolus	19
Nucleus	99
Organelle	198
Phragmoplast	5
Plasma membrane	69
Plasmodesma	21
Plastid	69
Proteasome complex	6
Proteasome core complex	5
Proteasome core complex, alpha-subunit complex	3
Protein complex	39
Ribonucleoprotein complex	13
Ribosome	25
Stromule	6
Vacuole	33

Molecular Function

2 iron, 2 sulfur cluster binding	5
Ammonia-lyase activity	2
ATP:ADP antiporter activity	2
Copper ion binding	14
Endopeptidase activity	5
Endoribonuclease activity	4
Hydrogen-translocating pyrophosphatase activity	2
Inositol phosphoceramide synthase activity	2
Iron-sulfur cluster binding	6
Lipid binding	11
L-malate dehydrogenase activity	3
Malate dehydrogenase activity	3
Mirna binding	3
Molecular_function	344
Oxidoreductase activity, acting on the CH-OH group of donors, NAD or NADP as acceptor	6
Phenylalanine ammonia-lyase activity	2
Phospholipid binding	5
Protein domain specific binding	2
RNA binding	27
Sirna binding	3
Structural constituent of ribosome	20
Structural molecule activity	25
Threonine-type endopeptidase activity	5
transcription factor binding	3

P-value 0.01

Supplementary Fig. S5. Gene Ontology enrichment analysis of genes targeted by miRNAs showing drought mediated downregulation in N22 flag leaf. The different GO terms with an enrichment pvalue of ≤ 0.01 belonging to biological process, molecular function and cellular component were shown with the number of genes and heat map representing the p-value.

Biological Process

Cellular response to UV-B	**2**
Cysteine biosynthetic process	**3**
Multicellular organismal development	**3**
Removal of superoxide radicals	**3**
Response to copper ion	**2**
Response to iron ion	**2**
Response to ozone	**2**
Response to salt stress	**8**
Stomatal lineage progression	**2**
Superoxide metabolic process	**3**
Ubiquitin-dependent protein catabolic process	**4**
Vegetative phase change	**2**
Virus induced gene silencing	**2**

Cellular Component

Cell	46
Chloroplast	14
Chloroplast envelope	7
Cytoplasm	32
Cytosol	12
Cytosolic large ribosomal subunit	3
Cytosolic ribosome	6
Intracellular	41
Membrane	20
Mitochondrial inner membrane	3
Nucleolus	6
Organelle	35
Plastid	19
Ribonucleoprotein complex	5
Ribosome	8

Molecular Function

Copper ion binding	14
Superoxide dismutase activity	3
Electron carrier activity	9
Antioxidant activity	2
Protein homodimerization activity	3
Phosphoprotein phosphatase activity	3
Structural constituent of ribosome	5

P-value 0.01

Supplementary Fig. S6. Gene Ontology enrichment analysis of genes targeted by miRNAs showing drought mediated up-regulation in N22 flag leaf. The different GO terms with an enrichment pvalue of ≤ 0.01 belonging to biological process, molecular function and cellular component were shown with the number of genes and heat map representing the p-value .

a

Biological Process

Adaxial/abaxial axis specification	2
adaxial/abaxial pattern specification	2
Biosynthetic process	35
Chlorophyll metabolic process	2
Determination of bilateral symmetry	4
Embryonic pattern specification	2
Integument development	2
Lignin biosynthetic process	2
Malate metabolic process	2
Meristem initiation	4
Polarity specification of adaxial/abaxial axis	4
Primary shoot apical meristem specification	2
Proteolysis involved in cellular protein catabolic process	2
Radial pattern formation	2
regulation of transcription, DNA-templated	14
transcription, DNA-templated	13
Translation	8
Xylem and phloem pattern formation	2

Cellular Component

Cell	62
Cellular_component	73
Chloroplast	17
Cytoplasm	39
Cytosol	20
Intracellular	54
Membrane	24
Nucleolus	7
Nucleus	30
Organelle	52
Plasma membrane	25
Plasmodesma	11
Plastid	18
Proteasome core complex	2
Ribonucleoprotein complex	7
Ribosome	7
Vacuolar membrane	7
Vacuole	10

Molecular Function

Copper ion binding	9
Electron carrier activity	8
Endopeptidase activity	2
Hydrolase activity, acting on acid anhydrides, in phosphorus-containing anhydrides	8
Lipid binding	5
Structural constituent of ribosome	7
Structural molecule activity	7
Threonine-type endopeptidase activity	2

b

Biological Process

Aging	4
Anatomical structure development	10
Biosynthetic process	18
Leaf senescence	2
Methionine biosynthetic process	2
regulation of transcription, DNA-templated	13
Response to cold	4
Response to salt stress	6
transcription, DNA-templated	7
Water transport	2

Cellular Component

Cell	34
Chloroplast envelope	6
Intracellular	31
Mitochondrial respiratory chain complex I	2
Mitochondrion	8
Organelle	27
Plastid	12

Molecular Function

2 iron, 2 sulfur cluster binding	2
DNA binding	14
Transporter activity	5

P-value 0.01

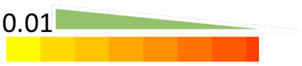

Supplementary Fig. S7. Gene Ontology enrichment analysis of genes targeted by miRNAs showing drought mediated (a) down and (b) up-regulation in PB1 flag leaf. The different GO terms with an enrichment pvalue of ≤ 0.01 belonging to biological process, molecular function and cellular component were shown with the number of genes and heat map representing the p-value.

a

Biological Process

Biosynthetic process	25
Catabolic process	18
Cellular amino acid biosynthetic process	3
Cellular amino acid metabolic process	7
Cellular nitrogen compound metabolic process	23
endonucleolytic cleavage in ITS1 to separate SSU-rRNA from 5.8S rRNA and LSU-rRNA from tricistronic rRNA transcript (SSU-rRNA, 5.8S rRNA, LSU-rRNA)	2
Endonucleolytic cleavage to generate mature 3'-end of SSU-rrna from (SSU-rrna, 5.8S rrna, LSU-rrna)	2
Nitrogen compound metabolic process	3
N-terminal protein myristoylation	2
Pentose-phosphate shunt	4
Peptidyl-proline modification	2
Photorespiration	3
Response to cadmium ion	7
Ribosomal small subunit biogenesis	2
Small molecule metabolic process	20
sulfur compound metabolic process	5
Translation	8
Translational elongation	4
Ubiquitin-dependent protein catabolic process	5

Cellular Component

Cell	57
Cellular_component	63
Chloroplast	18
Chloroplast envelope	7
Chloroplast thylakoid membrane	5
Cytoplasm	41
Cytoplasmic vesicle	2
Cytosol	17
Intracellular	52
Membrane	22
Organelle	44
Plasma membrane	18
Plastid	21
Protein complex	13
Thylakoid	6

Molecular Function

Coenzyme binding	3
Copper ion binding	6
FK506 binding	2
Hydrolase activity, acting on carbon-nitrogen (but not peptide) bonds	3
Isomerase activity	5
Ligase activity	6
Racemase and epimerase activity, acting on carbohydrates and derivatives	2
Structural molecule activity	6
Translation elongation factor activity	2

b

Biological Process

Biosynthetic process	9
Transcription, DNA-templated	5
Stomatal lineage progression	2

Cellular Component

Cytosol	5
Intracellular	13

Molecular Function

Transcription factor binding	2
DNA binding	7
Protein dimerization activity	3
ATP binding	7
Helicase activity	3

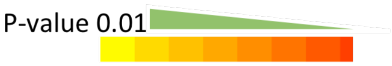

Supplementary Fig. S8. Gene Ontology enrichment analysis of genes targeted by miRNAs showing drought mediated (a) down and (b) up-regulation in N22 spikelet. The different GO terms with an enrichment pvalue of ≤ 0.01 belonging to biological process, molecular function and cellular component were shown with the number of genes and heat map representing the p-value.

a

b

(a)

Biological Process

Anthocyanin-containing compound metabolic process		2
ATP biosynthetic process		2
Biosynthetic process		35
Catabolic process		17
Cellular amino acid metabolic process		9
Cysteine biosynthetic process		3
Defense response to bacterium		5
Endonucleolytic cleavage in ITS1 to separate SSU-rrna from 5.8S rrna and LSU-rrna from tricistronic rrna transcript		2
Endonucleolytic cleavage to generate mature 3'-end of SSU-rrna from (SSU-rrna, 5.8S rrna, LSU-rrna)		2
Regulation of ARF gtpase activity		2
Ribosomal small subunit biogenesis		2
Salicylic acid biosynthetic process		2
Small molecule metabolic process		22
Translation		11
Translational elongation		5
Ubiquitin-dependent protein catabolic process		6

Cellular Component

Cell		63
Cellular_component		71
Chloroplast		24
Chloroplast envelope		9
Cytoplasm		43
Cytosol		18
Cytosolic ribosome		6
Cytosolic small ribosomal subunit		3
Intracellular		56
Organelle		48
Plastid		25
Ribonucleoprotein complex		6
Ribosome		8

Molecular Function

Acid-amino acid ligase activity		4
ARF gtpase activator activity		2
Copper ion binding		10
Electron carrier activity		9
Ligase activity		8
Phosphotransferase activity, alcohol group as acceptor		3
Structural constituent of ribosome		8
Structural molecule activity		8

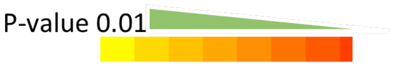

(b)

Biological Process

ATP synthesis coupled proton transport		2
auxin-activated signaling pathway		3
Biosynthetic process		25
Calcium-mediated signaling		2
Cellular component assembly		6
Cellular protein metabolic process		2
Cellular response to nitrate		2
Cellular response to phosphate starvation		3
Iron ion homeostasis		2
Lateral root development		2
Lateral root formation		2
Macromolecular complex assembly		6
Pollen maturation		2
Protein complex assembly		5
Protein folding		4
Protein targeting to chloroplast		2
Regulation of photomorphogenesis		2
Response to cadmium ion		10
Response to molecule of bacterial origin		2
Response to salt stress		7
Stamen development		2

Cellular Component

Apoplast		7
Cell		54
Cell wall		8
Cellular_component		60
Chloroplast stroma		7
Cytoplasm		39
Cytosol		17
Extracellular region		7
Intracellular		49
Membrane		20
Mitochondrial respiratory chain complex I		3
Mitochondrion		11
Nucleolus		6
Nucleus		19
Organelle		43
Plasma membrane		22
Plasmodesma		8
Protein complex		12
SCF ubiquitin ligase complex		2
Vacuolar membrane		6
Vacuole		8

Molecular Function

Auxin binding		2
Inositol hexakisphosphate binding		2
Transcription factor binding		2

Supplementary Fig. S9. Gene Ontology enrichment analysis of genes targeted by miRNAs showing drought mediated (a) down and (b) up-regulation in PB1 spikelet. The different GO terms with an enrichment pvalue of ≤ 0.01 belonging to biological process, molecular function and cellular component were shown with the number of genes and heat map representing the p-value.

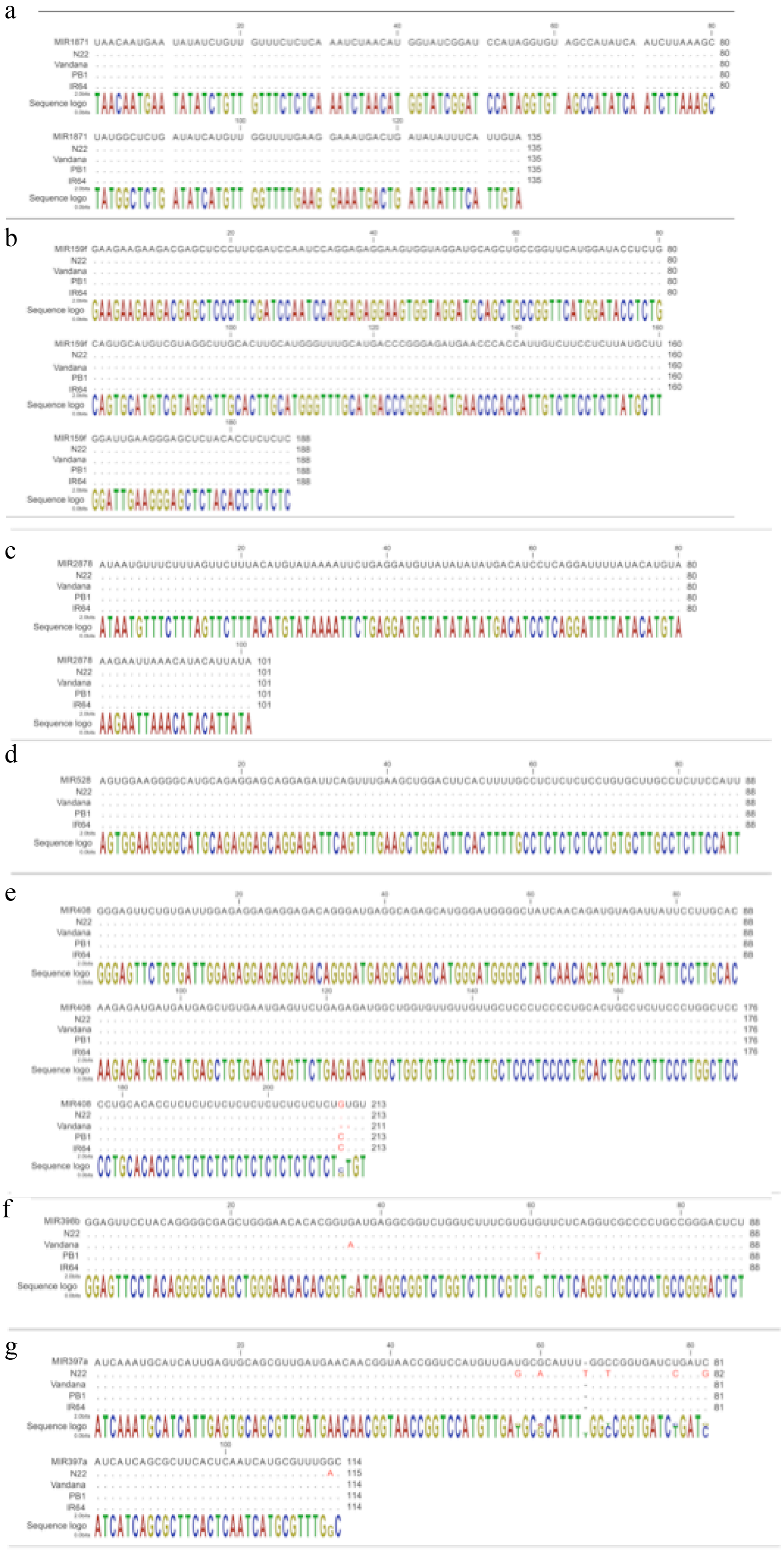

Supplementary Fig. S10. Multiple sequence alignment of MIR-precursor sequences from N22, PB1, Vandana and IR64. (**a**) MIR1871 precursor; (**b**) MIR159f; (**c**) MIR2878; (**d**) MIR528; (**e**) MIR408; (**f**) MIR398b and (**g**) MIR397a. Dots (.) represents the identical sequence bases and SNPs are shown in red letters.

a.

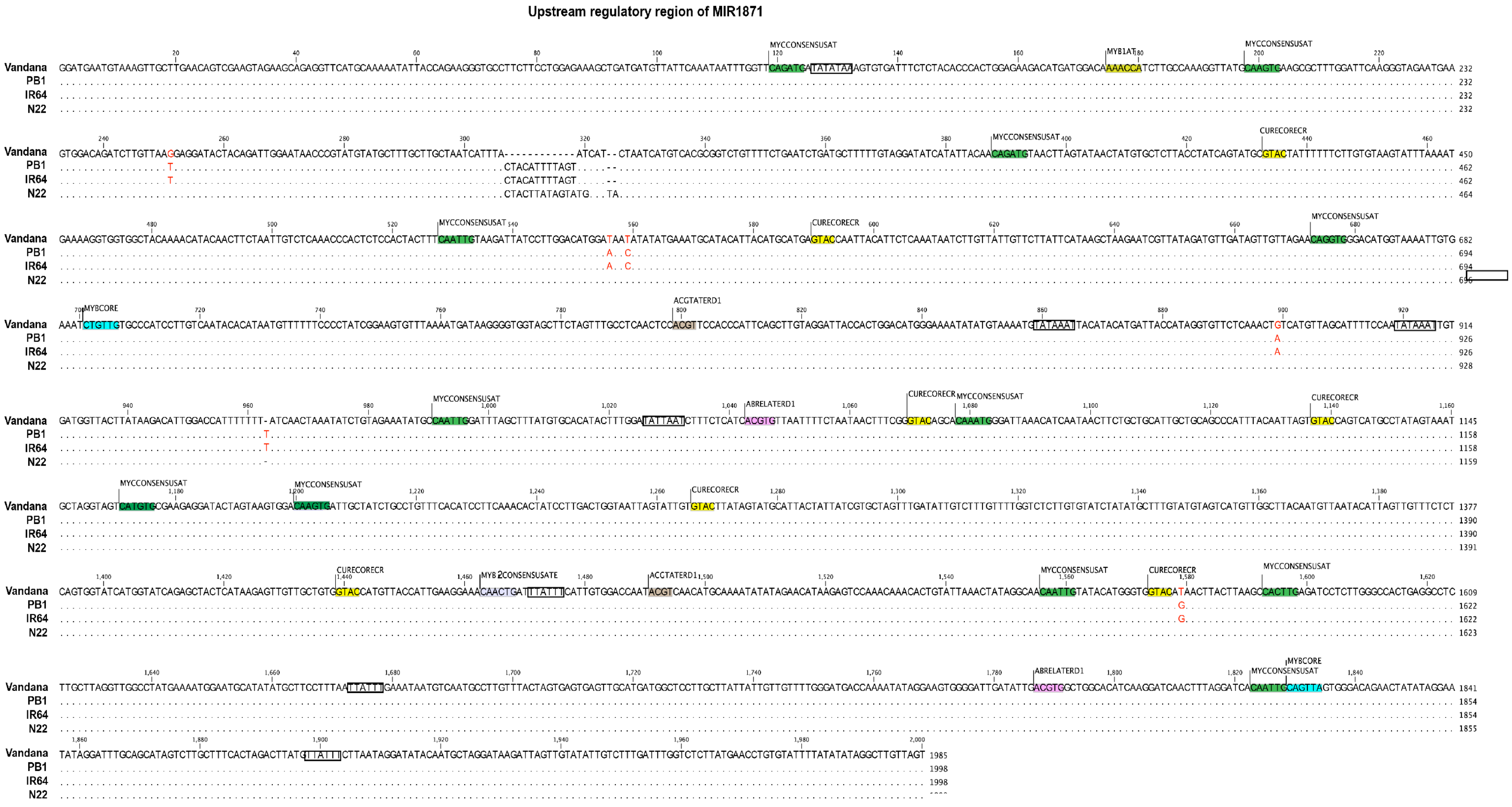

b.

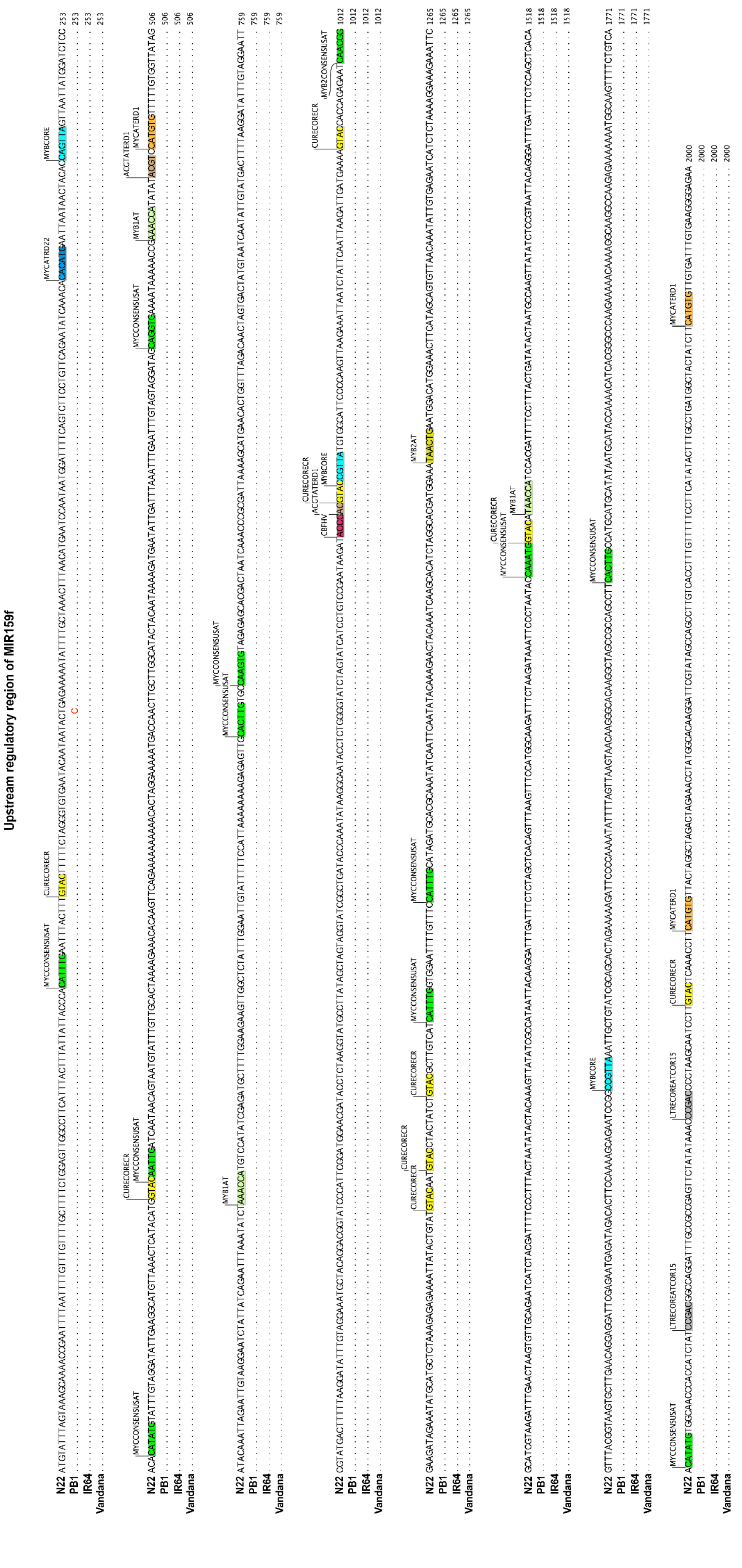

c.

Upstream regulatory region of MIR2878

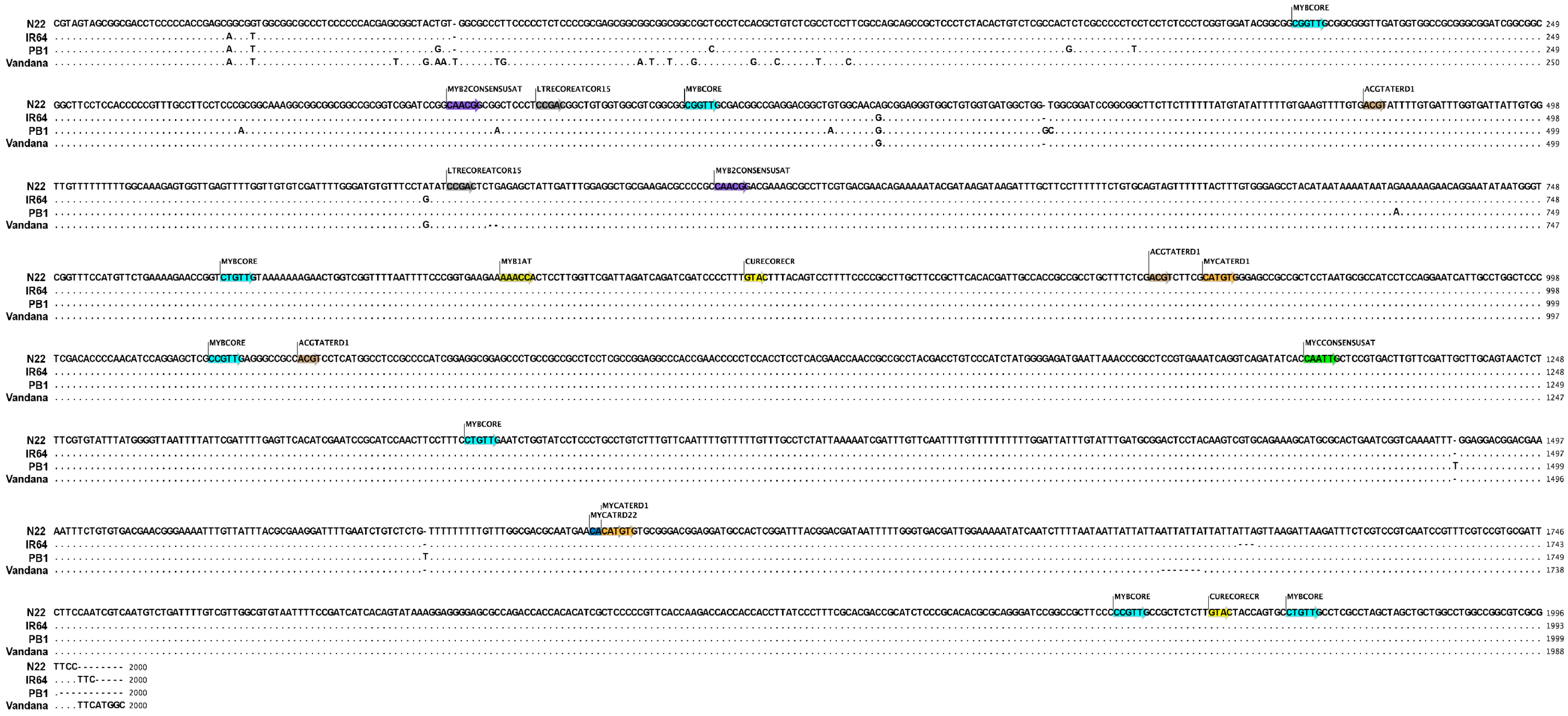

d.

Upstream regulatory region of MIR528

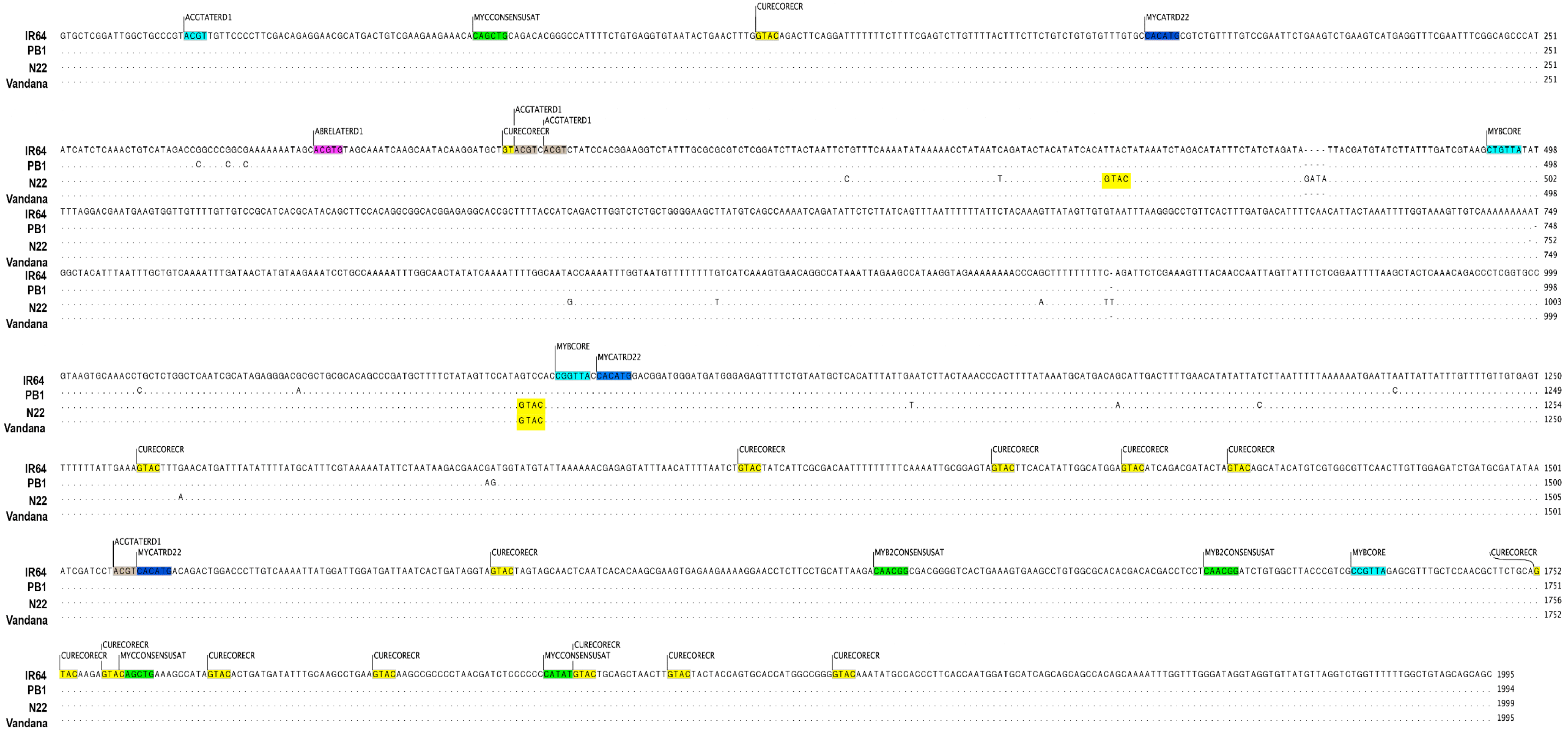

e.

Upstream regulatory region of MIR408

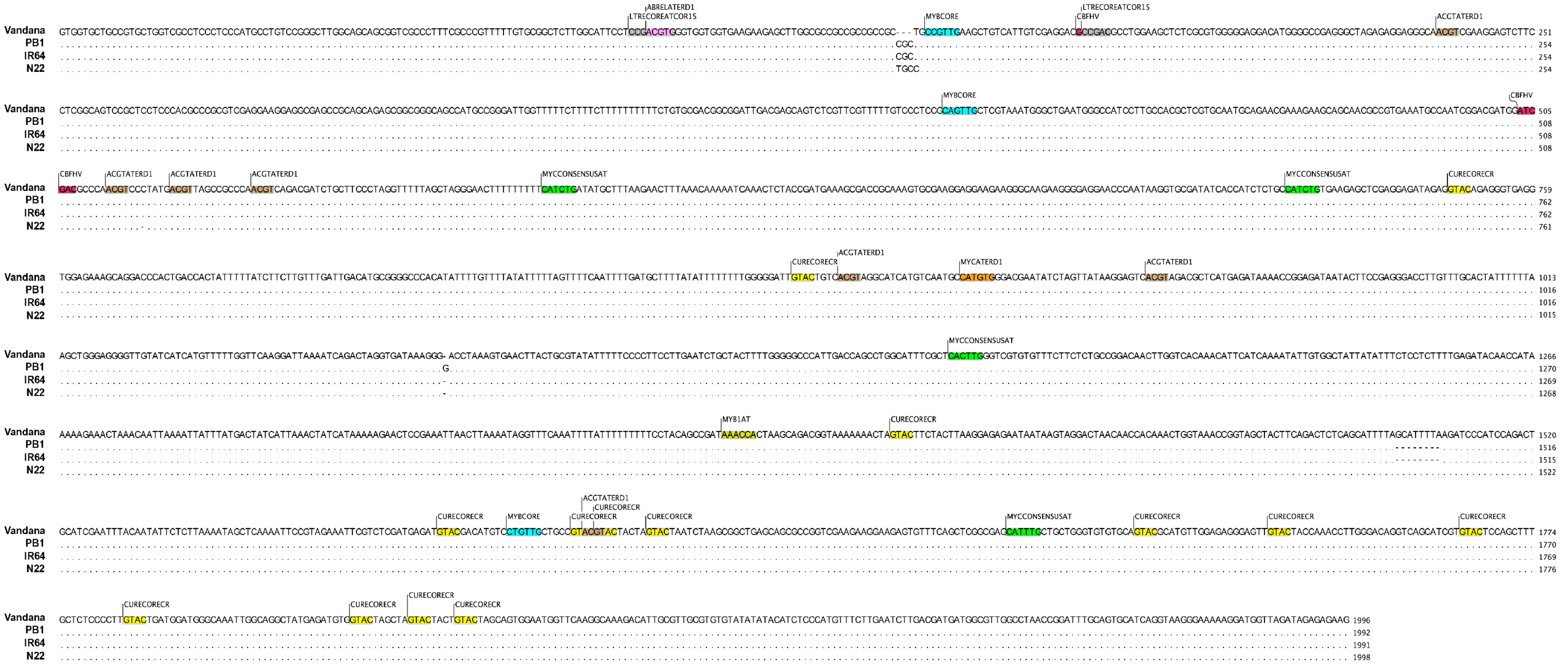

f.

Upstream regulatory region of MIR398b

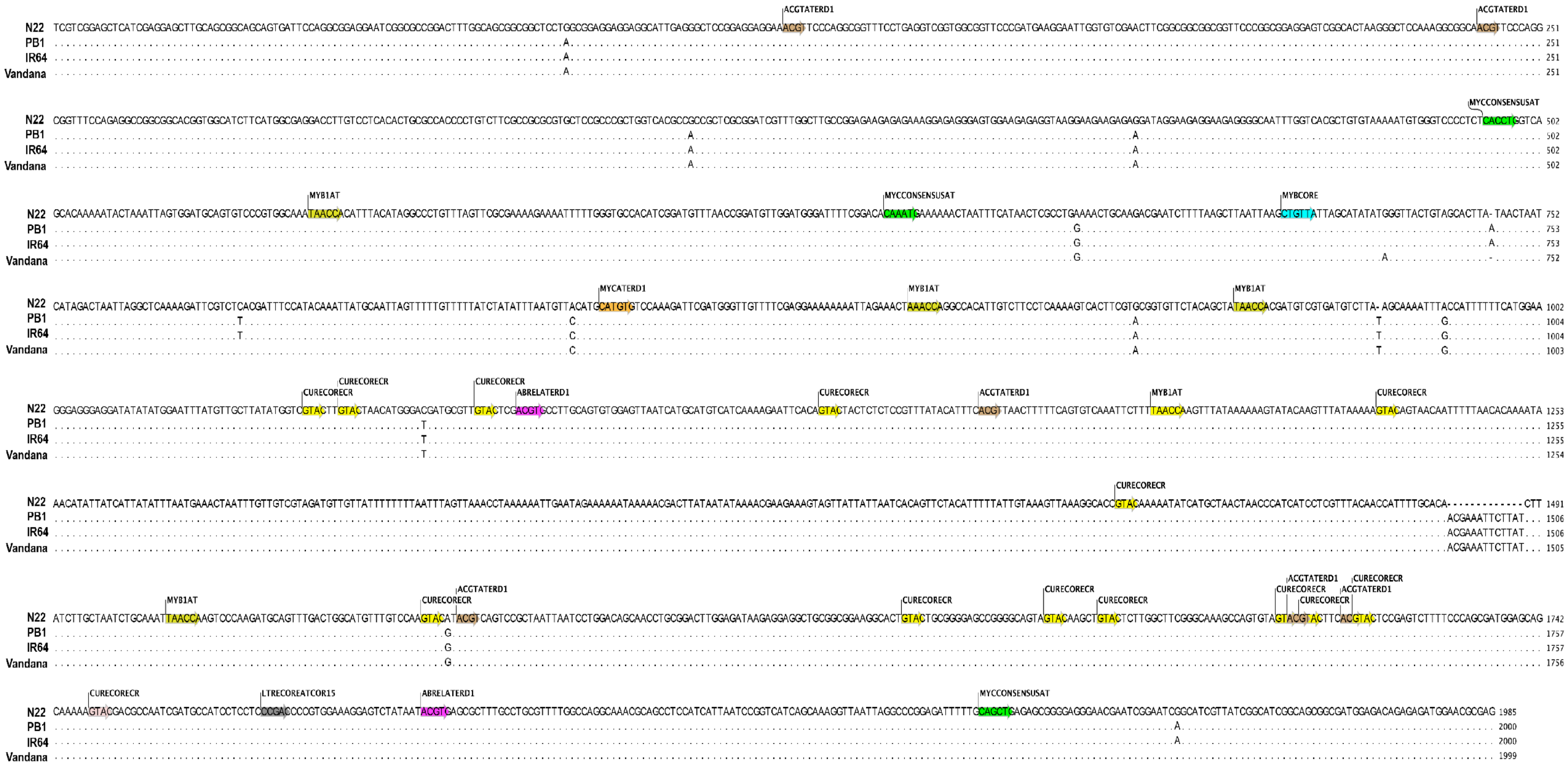

g.

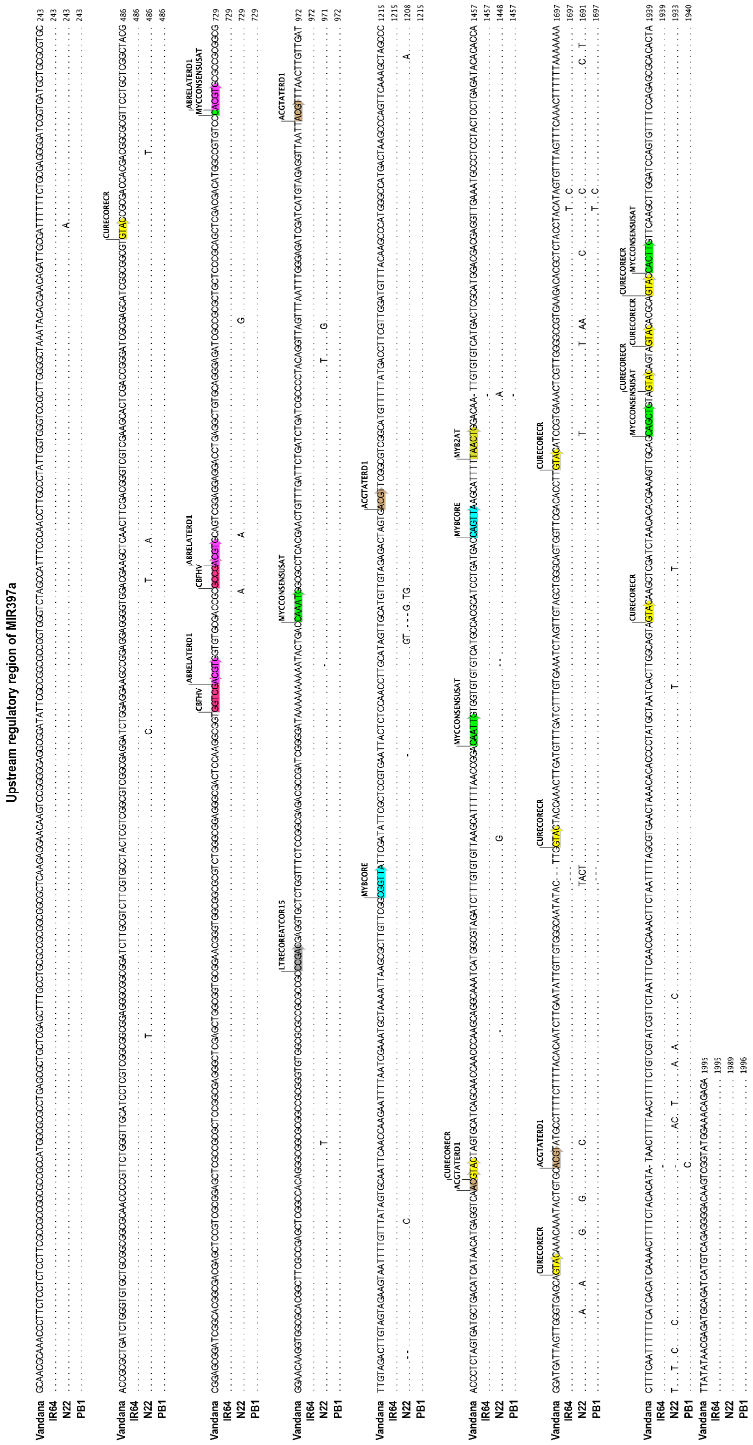

Supplementary Fig. S11. Sequence alignment of DTA-miRNA promoter sequences in N22, Vandana, PB1 and IR64. Sequence alignment of 2kb upstream region of (**a**) MIR1871, (**b**) MIR159f 2kb, (**c**) MIR2878, (**d**) MIR528, (**e**) MIR408, (**f**) MIR398b, (**g**) MIR397a in four cultivars namely N22, Vandana, PB1 and IR64. Dots (.) represents the identical sequence, dashes (-) represents gaps and the cis-acting elements associated with stress were highlighted.

a

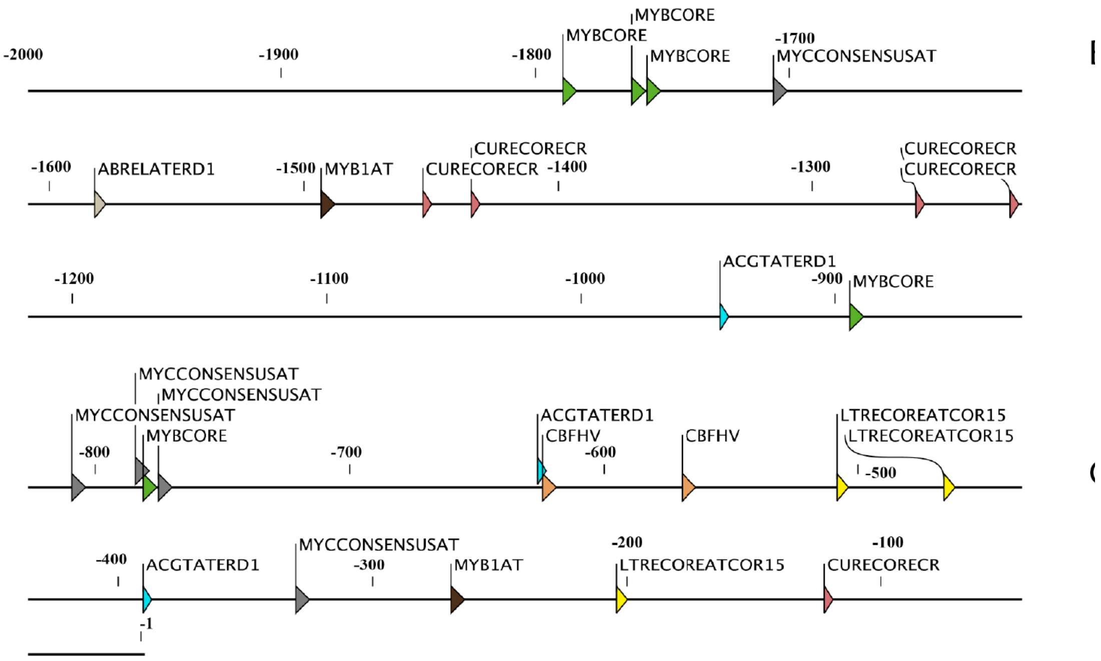

b

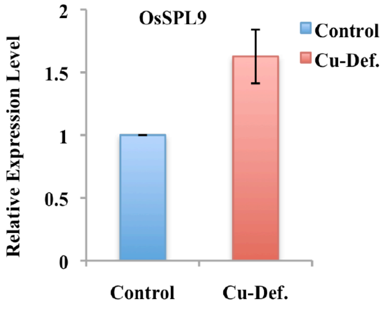

Supplementary Fig. S12. OsSPL9 promoter analysis (**a**) Cis-regulatory elements in the 2kb upstream promoter of OsSPL9. (**b**) RT-PCR of OsSPL9 in response to copper starvation in N22 seedlings. Data represents the mean value of three biological replicates and three technical repeats.

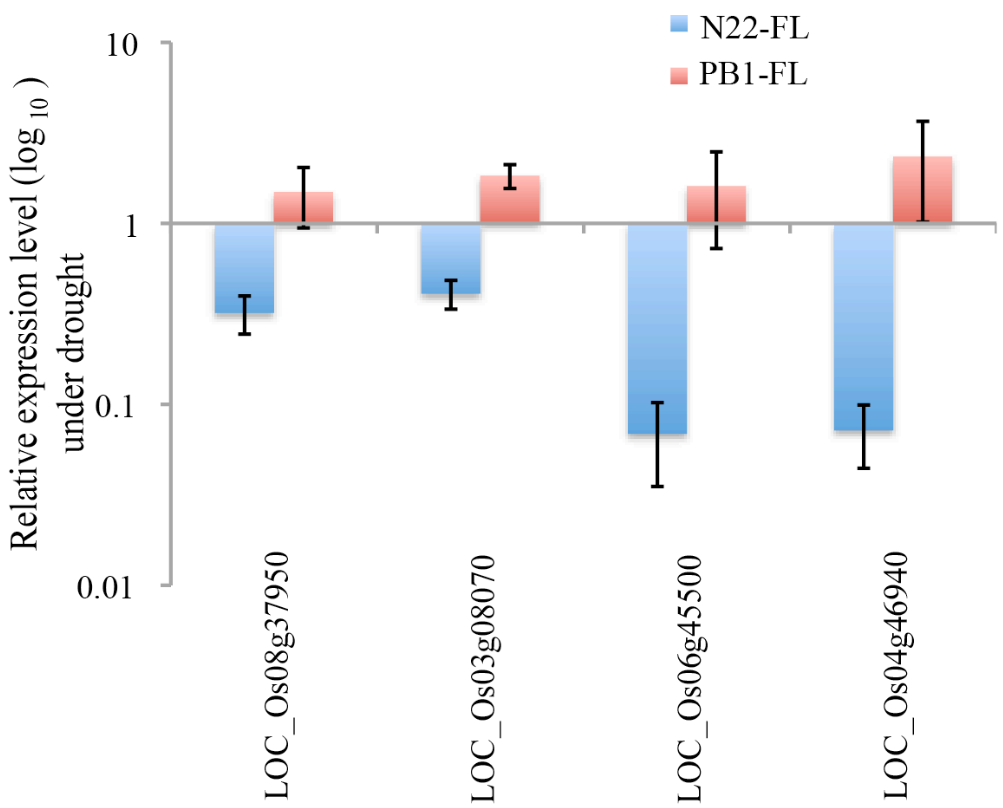

Supplementary Fig. S13. qRT-PCR of copper ATPases in N22 and PB1 flag leaf in response to drought. Three biological replicates were analyzed with three technical repeats. Error bars represent the standard error.

a COPT1

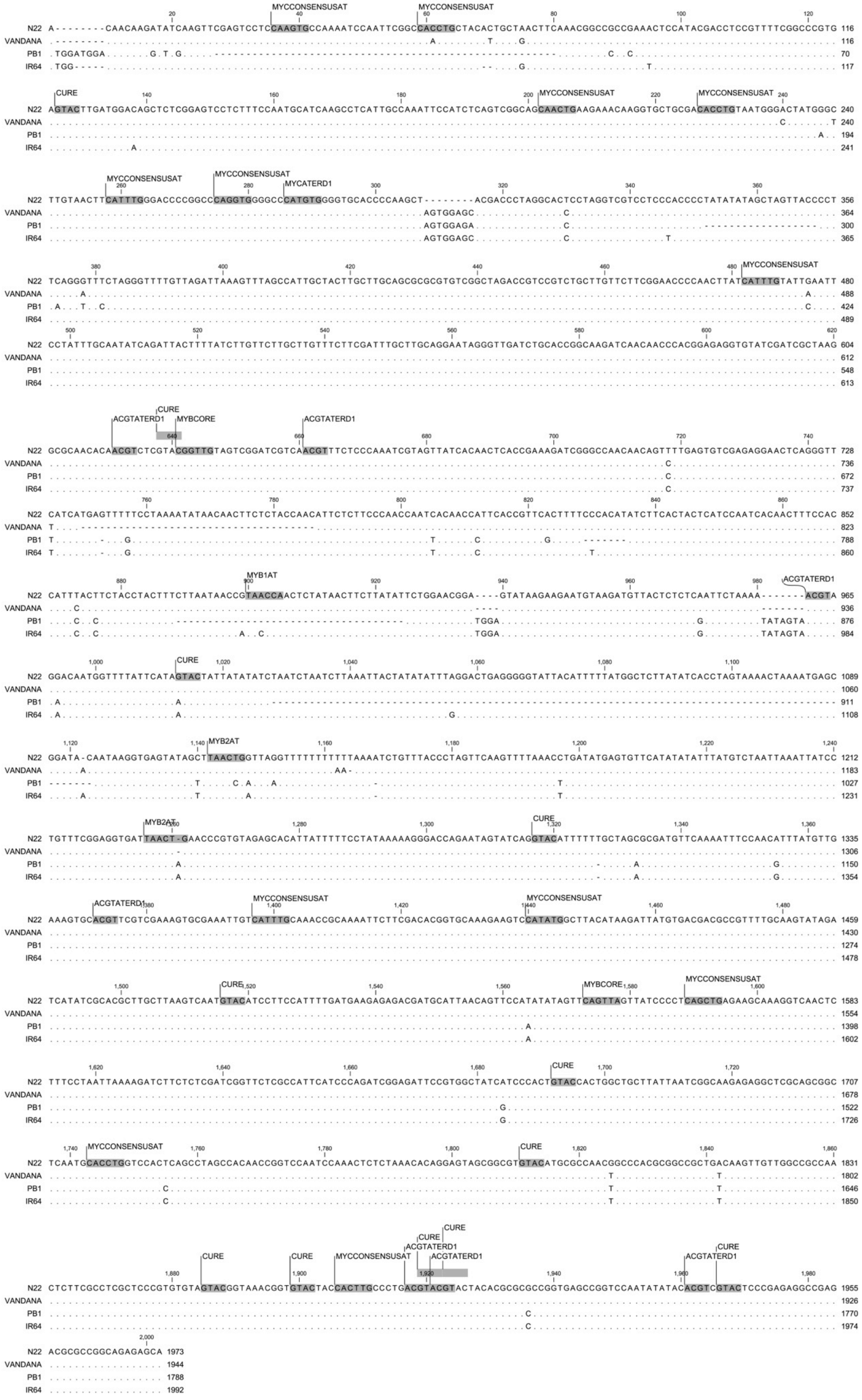
N22
VANDANA
PB1
IR64
MYCCONSENSUSAT
CURE
MYCATERD1
ACGTATERD1
MYBCORE
MYB1AT
MYB2AT

b COPT2

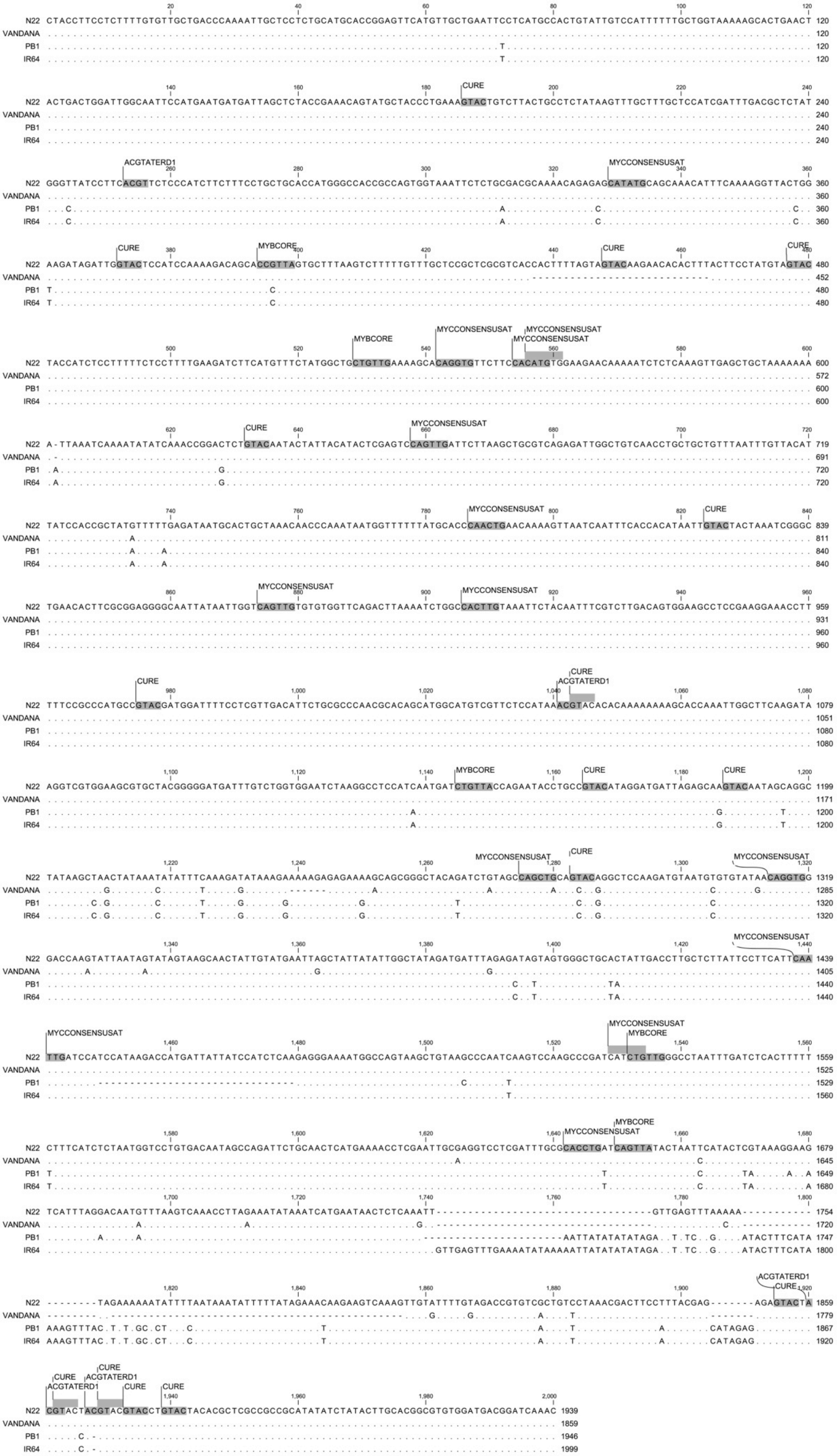
N22
VANDANA
PB1
IR64
CURE
ACGTATERD1
MYCCONSENSUSAT
MYBCORE

c COPT3

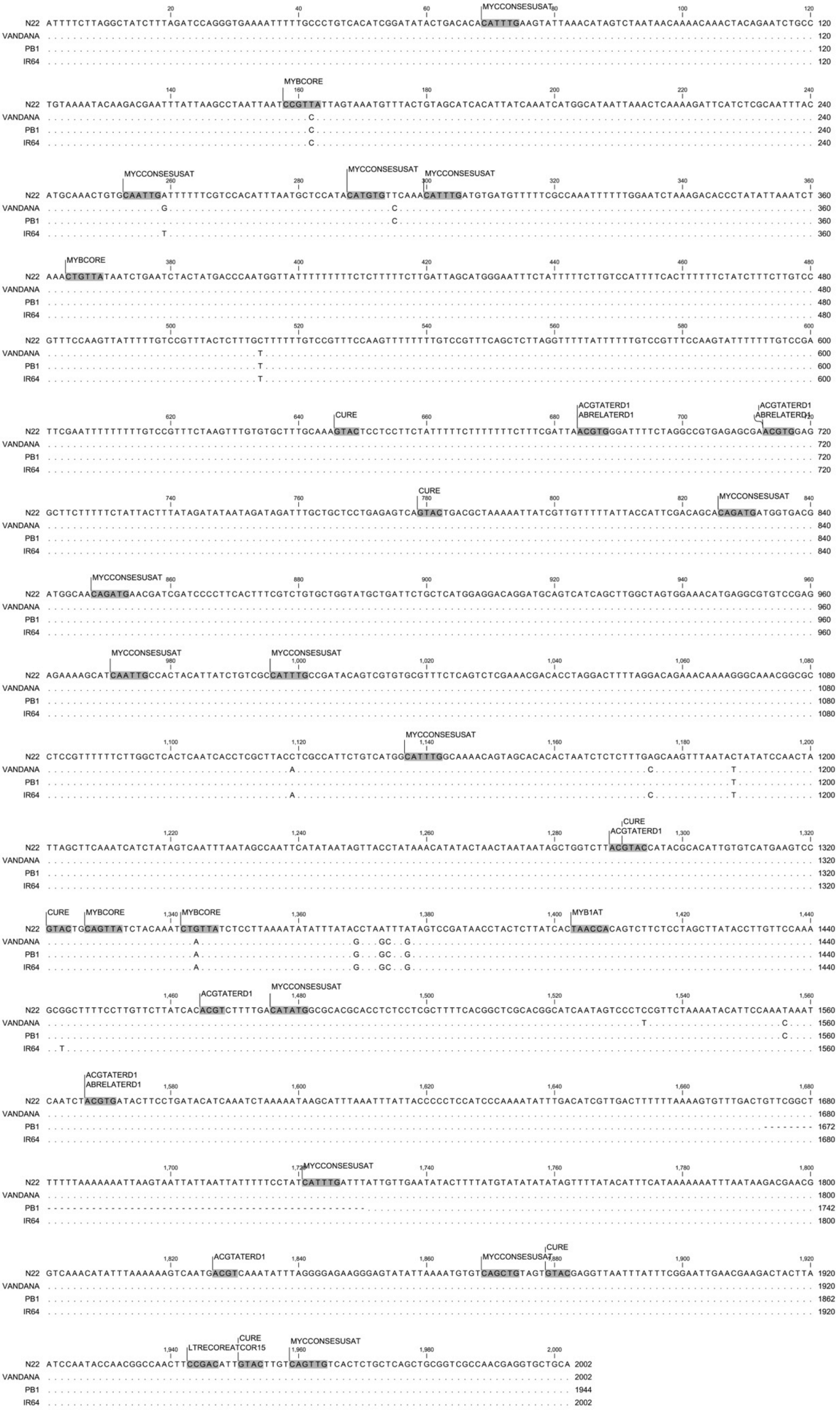
N22
VANDANA
PB1
IR64
MYCCONSESUSAT
MYBCORE
CURE
ACGTATERD1
ABRELATERD1
MYB1AT
LTRECOREATCOR15

d COPT4

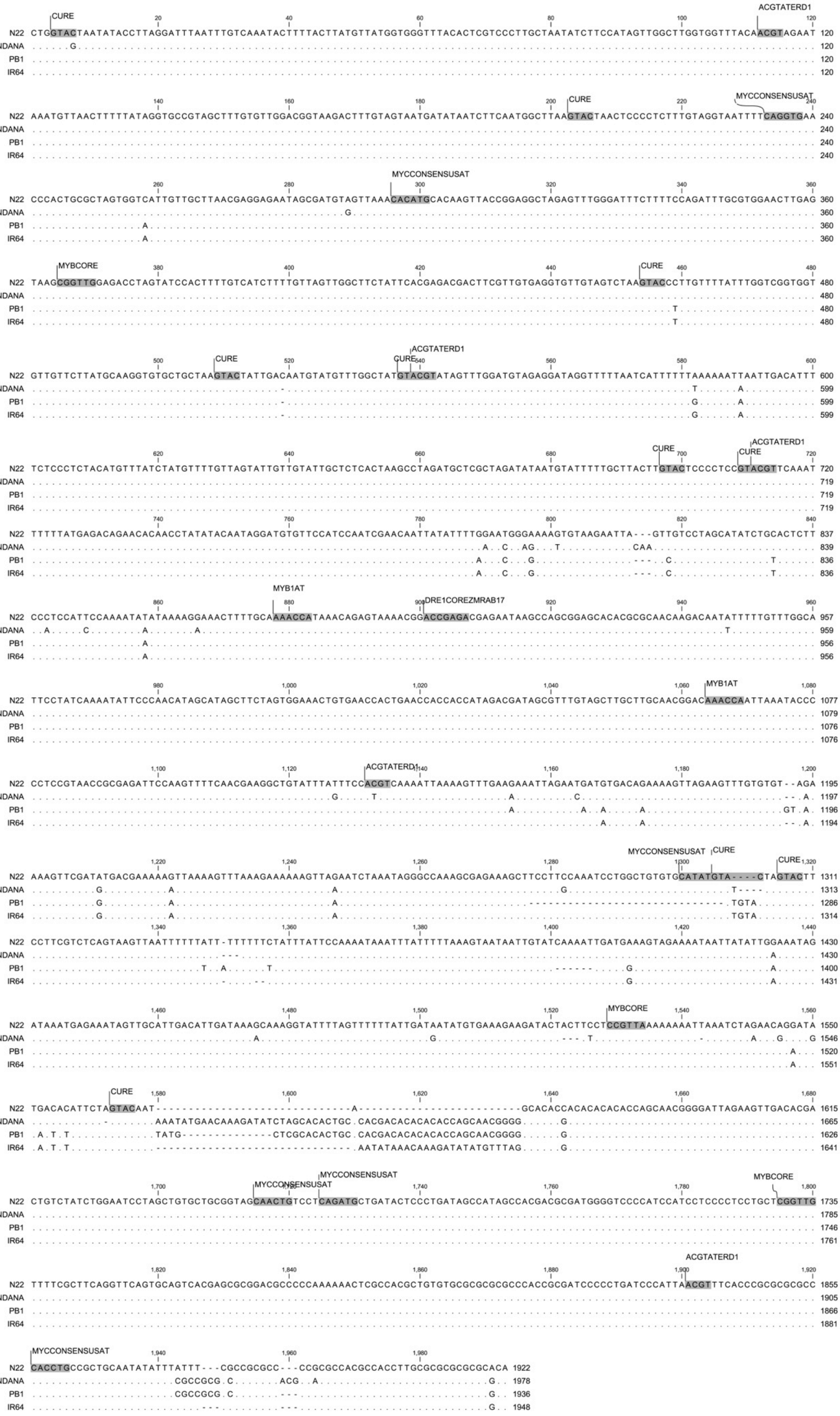

N22
VANDANA
PB1
IR64
CURE
ACGTATERD1
MYCCONSENSUSAT
MYBCORE
MYB1AT
DRE1COREZMRAB17

e COPT5

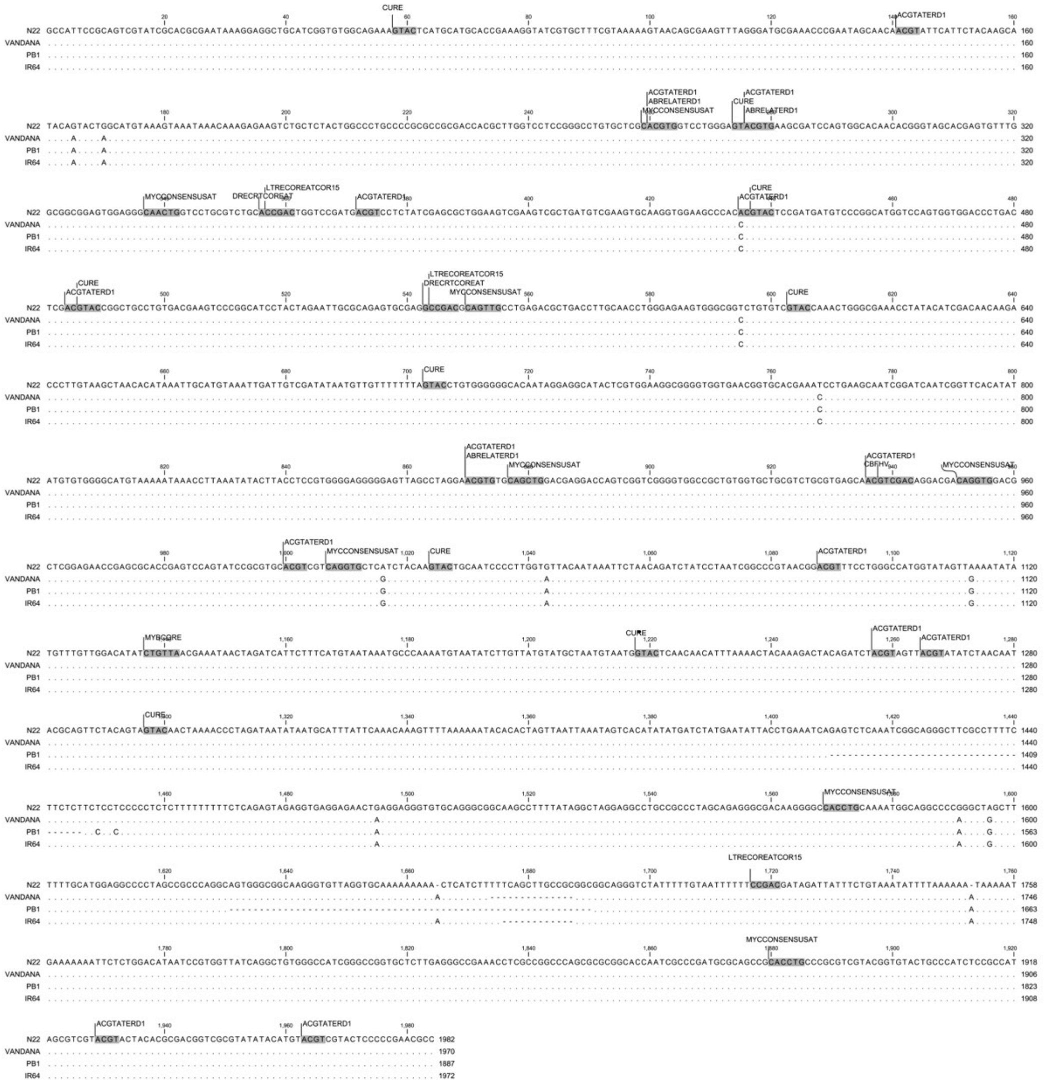

f COPT6

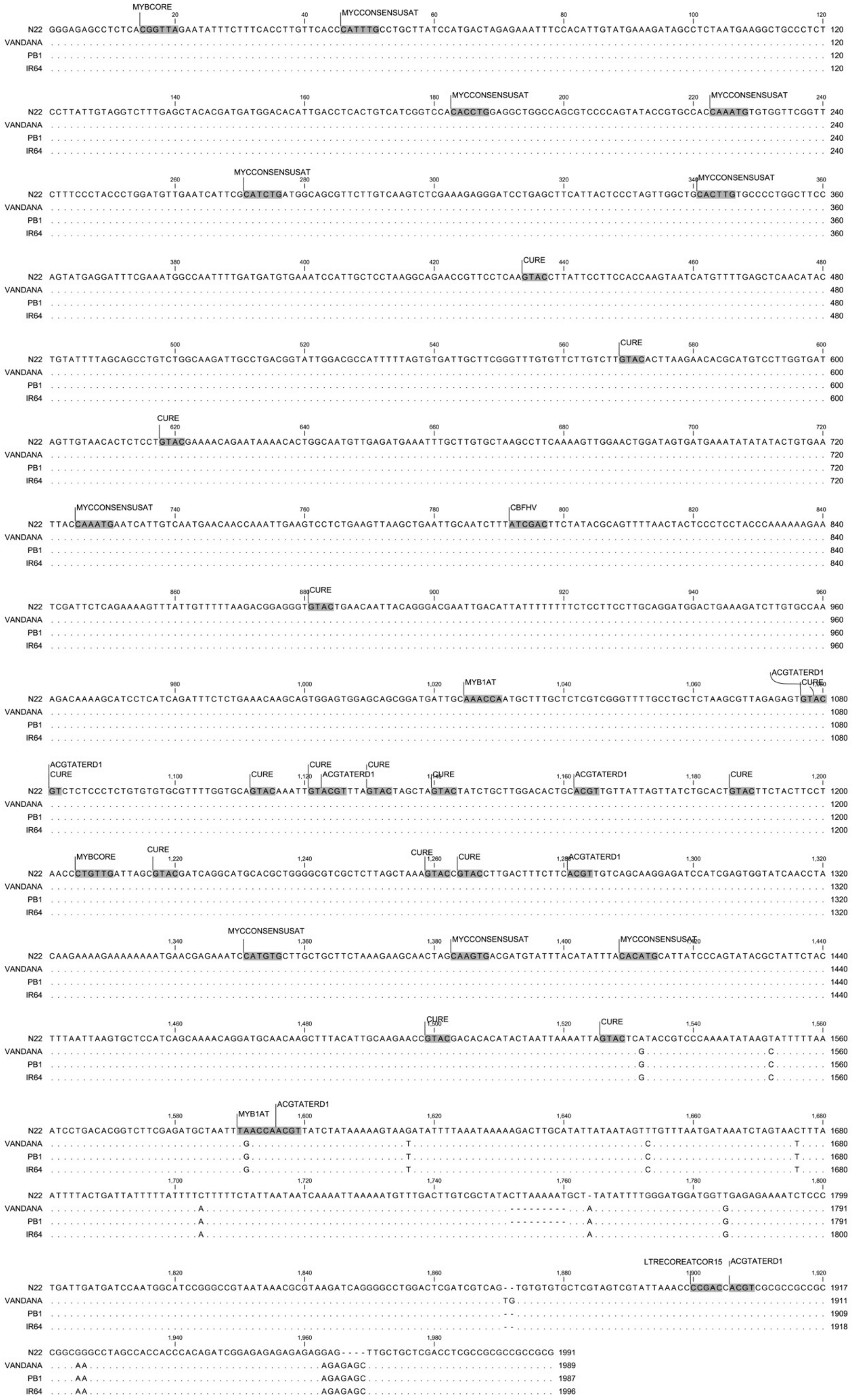
N22
VANDANA
PB1
IR64
MYBCORE
MYCCONSENSUSAT
CURE
CBFHV
MYB1AT
ACGTATERD1
LTRECOREATCOR15

g COPT7

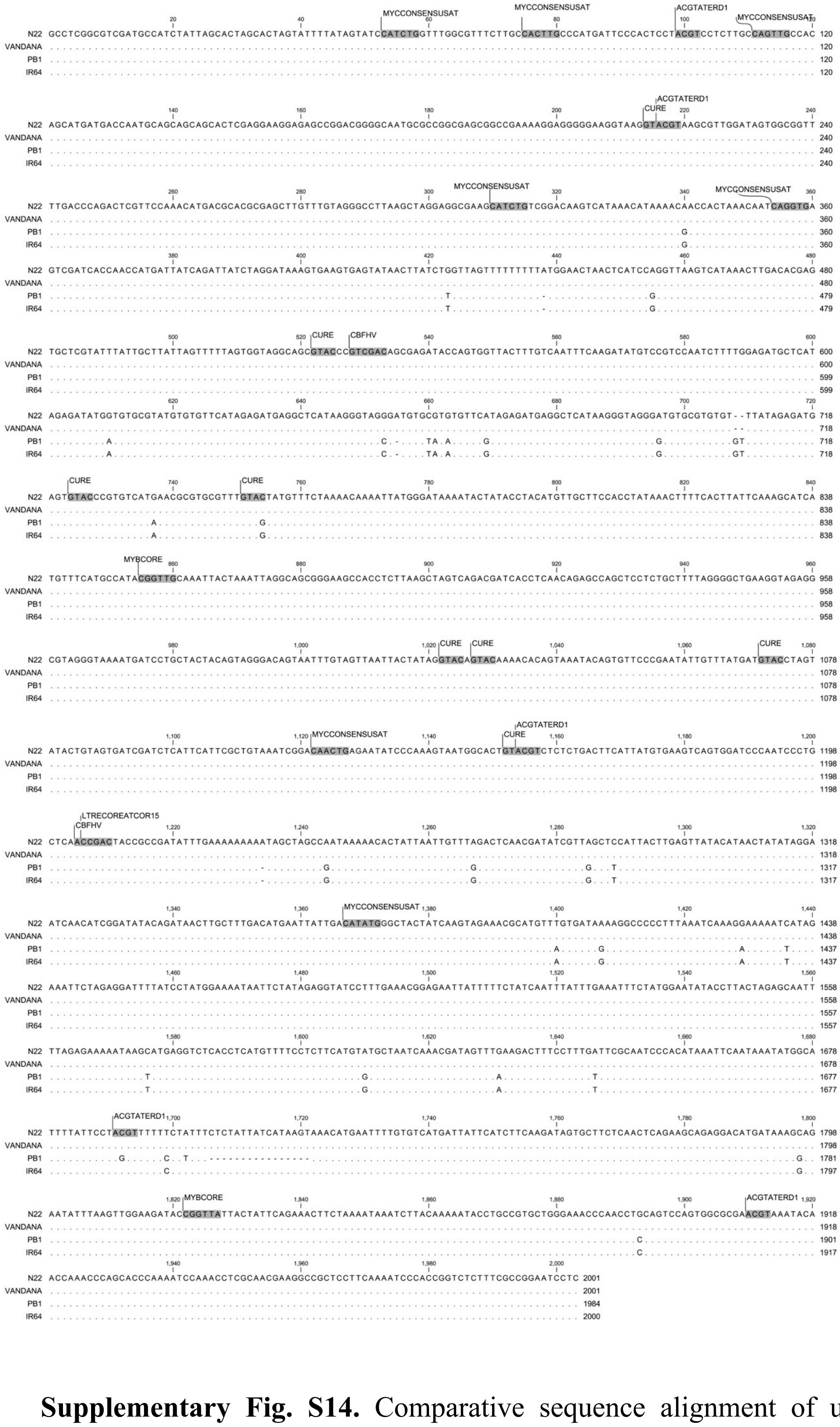

Supplementary Fig. S14. Comparative sequence alignment of upstream regulatory region of the seven rice copper transporters. Sequence alignment of 2kb upstream region of (**a**) COPT1, (**b**) COPT2, (**c**) COPT3, (**d**) COPT4, (**e**) COPT5, (**f**) COPT6, (**g**) COPT7 in four cultivars namely N22, Vandana, PB1 and IR64. Dots (.) represents the identical sequence, dashes (-) represents gaps and the cis-acting elements associated with stress were highlighted

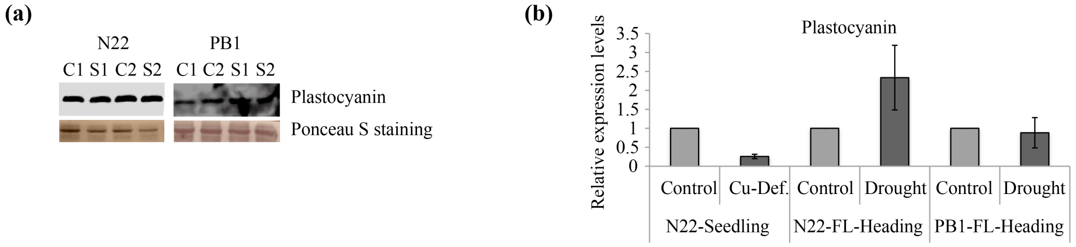

Supplementary Fig. 15. Differential copper homeostasis does not modulate plastocyanin levels but affects ROS homeostasis. (**a**) Quantitative immunoblot showing the plastocyanin protein levels in N22 and PB1 flag leaf under control (C) and drought stress (S) conditions. Ponceau S staining was used to show the equal loading of samples. (**b**) Relative expression analysis of plastocyanin in N22 seedlings under copper starvation and in N22 and PB1 flag leaf under drought. Three biological with three technical replicates were analyzed.

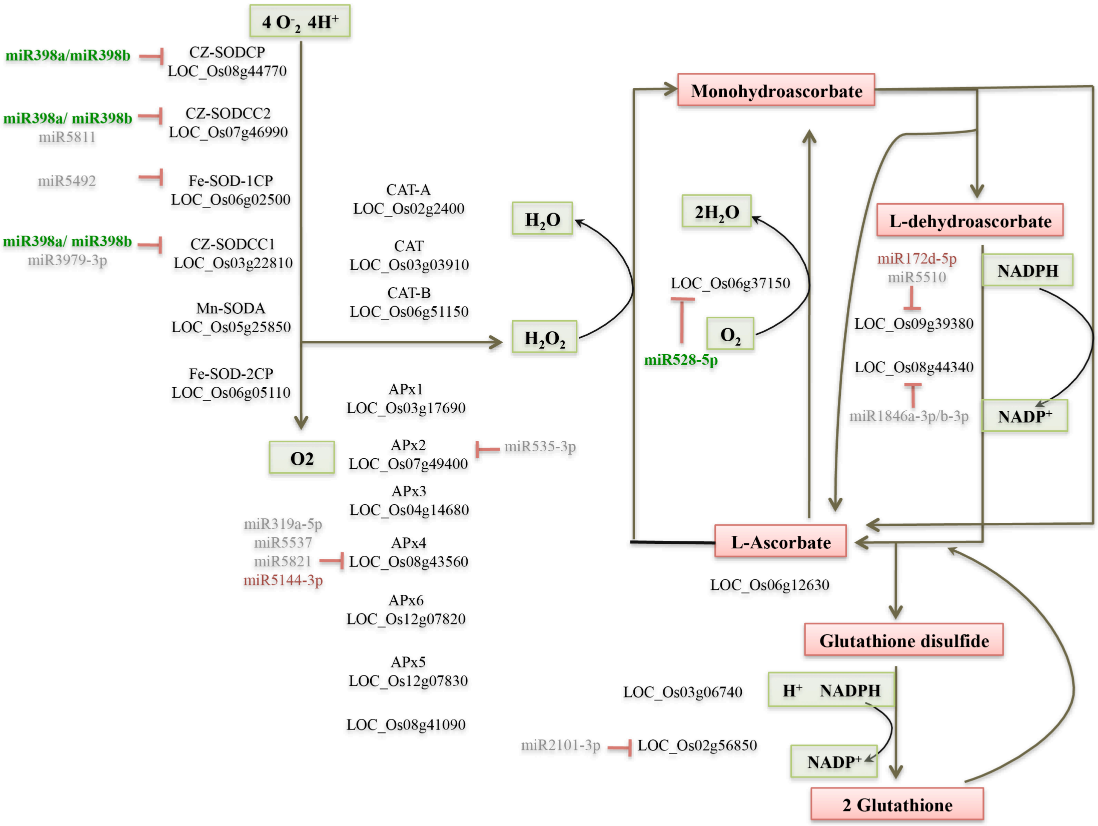

Supplementary Fig. S16. miRNA-mediated regulation of the pathways involved in the acquisition of ROS detoxification of ROS through removal of superoxide ions and ascorbate glutathione cycle. All genes were searched for putative targeting miRNA in the PARE data and the miRNA in green are significantly upregulated in N22 flag leaf while downregulated in PB1 flag leaf. miRNA in red are low in abundance but downregulated in N22 flag leaf and upregulated in PB1 flag leaf.

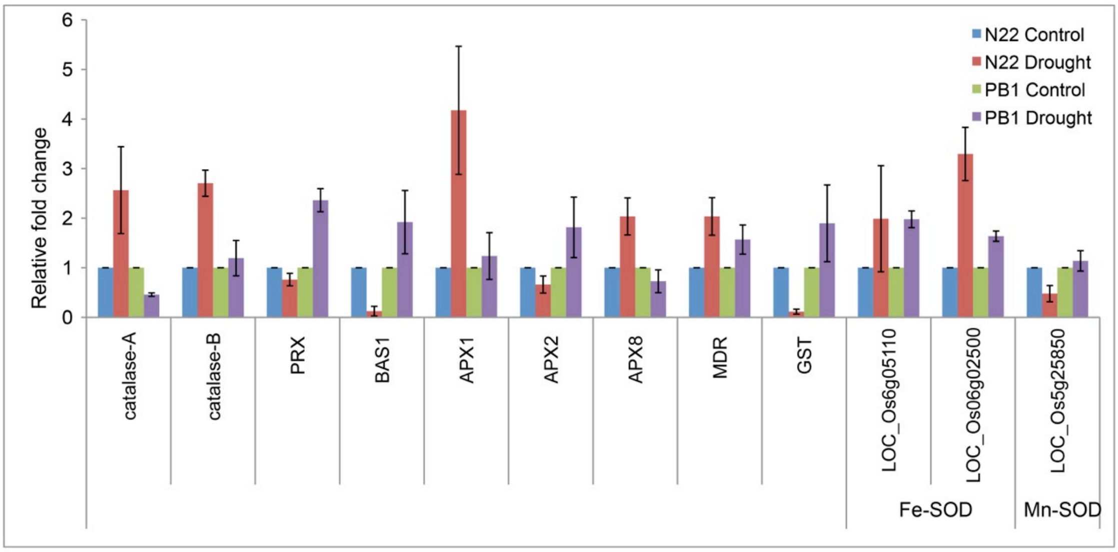

Supplementary Fig. S17. Expression analysis of genes involved in ROS homeostasis. qRT-PCR analysis of genes involved in the maintenance of ROS homeostasis in the flag leaf of N22 and PB1 under drought. Data represents the analyzed value of at least three biological and technical replicate. Error bars represents the standard error.

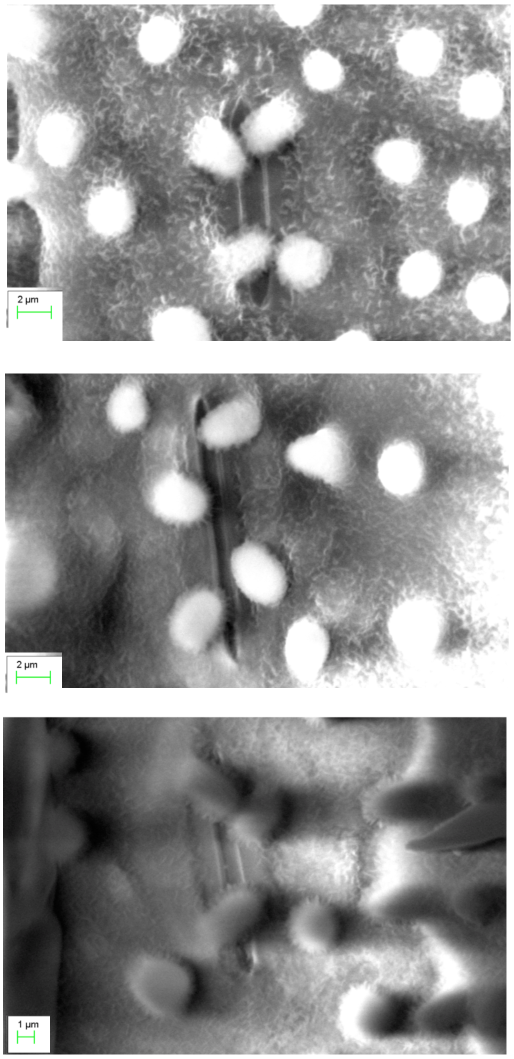

Open stomata

Partially-open stomata

Completely closed stomata

Supplementary Fig. S18. Electron micrographs showing the completely open, partially open and closed stomata in PB1 flag leaf.
